# Assessing Structural and Optical Properties of PTQ10-Based Donor Polymers in Solution for Eco-Friendly Photovoltaics: A Multiscale Modeling Study

**DOI:** 10.1021/acs.jpcb.5c01972

**Published:** 2025-05-30

**Authors:** Rafael B. Ribeiro, Leandro R. Franco, Alexandre Holmes, Tárcius N. Ramos, Ergang Wang, Márcio T. do N. Varella, C. Moyses Araujo

**Affiliations:** † Institute of Physics, University of São Paulo, Rua do Matão 1731, 05508-090 São Paulo, São Paulo, Brazil; ‡ Materials Theory Division, Department of Physics and Astronomy, 8097 Uppsala University, 75120 Uppsala, Sweden; § Department of Engineering and Physics, Karlstad University, 65188 Karlstad, Sweden; ∥ Department of Chemistry and Chemical Engineering, 11248 Chalmers University of Technology, SE-412 96 Göteborg, Sweden; ⊥ Theoretical Chemistry Lab, Unit of Theoretical and Structural Physical Chemistry, Namur Institute of Structured Matter, University of Namur, Rue de Bruxelles, 61, B-5000 Namur, Belgium

## Abstract

Aqueous-processable materials are desired to produce and commercialize eco-friendly organic solar cells. Despite the achievement of developing aqueous soluble electron donor and acceptor polymers by incorporating polar side chains (SCs), the efficiency of the greenest devices is lower than that of state-of-the-art technology processed on halogenated solvents. To investigate the impact of different substituents on structural and optical properties in solution, we considered the backbone of the PTQ10 polymer with alkyl and alkoxy SCs. We simulated oligomer chains at low and high concentration conditions via classical molecular dynamics simulations, considering both a water/ethanol mixture and chloroform as solvents. Combining an unsupervised machine learning technique and density functional theory calculations, we validated the system size for quantum calculations and investigated the impact of SCs on the excited states. Then, following the sequential QM/MM approach, we determined the absorption spectra of each polymer. From the simulations at high concentrations, we observed the stacking of different oligomers, suggesting that polymer chains already showed aggregation in solution. This is consistent with our experimental findings, as we measured a red shift of the PTQ­(8bO2) spectrum when changing from a chloroform mixture to an aqueous mixture. Finally, we investigated idealized dimer interface models, whose presence of electron-donating and electron-accepting groups results in mixed signatures in the absorption spectra, widening our understanding of polymer aggregation.

## Graphical abstract

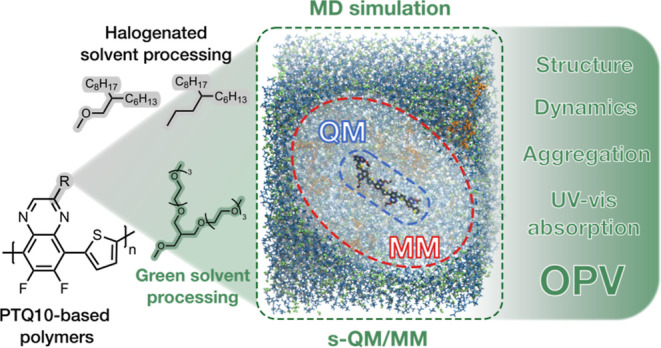

## Introduction

Photovoltaic technologies have been a primary candidate for replacing fossil fuels in the race for renewable and eco-friendly energy production.^1–3^ In particular, the development of new organic materials has significantly contributed to improve the performance of emerging solar cells.^4–9^ Due to their flexible design, organic molecules can be efficiently synthesized to match desirable structural and optoelectronic properties with typically lower production costs than inorganic materials.^10,11^ Promising devices have been developed by combining organic and inorganic semiconductors^12–14^ but with considerable downgrades, such as high sensitivity toward defects and the presence of heavy metals (e.g., in perovskite solar cells). Alternatively, purely organic solar cells (OSCs) showed significant growth in the past years, surpassing 20% of power conversion efficiency (PCE) with the development of the Y-shaped family of electron acceptors.^15–19^ Among the several layers that comprise such devices, the active layer of highly efficient OSCs, where the light-absorbing materials are arranged to start the energy conversion process, is typically produced via solution-processing techniques that rely on organic solvents. Despite the remarkable performances, large-scale production is impeded by such volatile and toxic solvents, requiring challenging conditions to avoid risks linked to leakage, exposure, and waste treatment.^20–22^ Additionally, most organic solvents are environmentally toxic and pose health risks,^23^ directly impacting the commercialization of eco-friendly OSCs.^24^ Therefore, designing new light-absorbing materials that are soluble in green solvents, like water, is important to overcome these undesired effects.

A common approach to designing aqueous-processable organic materials involves substituting alkylated to glycolated side chains (SCs) and adding polar groups such as triethylene glycol (TEG) and oligo­(ethylene glycol) (OEG). The addition of oxygens introduces significant hydrophilicity and polar interactions, enabling better solubility in green solvents without compromising the optoelectronic properties dictated by the backbone structure.^25^ However, such modification has a severe impact on the energy conversion performance. Since the novel development of OEG-based aqueous-processable conjugated polymers by Woo et al.^26^ in 2017, the most efficient devices combine ethanol and water mixtures to achieve PCE values close to 3%.^27–29^ Among the best-performing all-polymer aqueous-processable OSCs, Wang et al.^30^ recently developed a family of quinoxaline (Qx)-based electron donor polymers with the most promising results achieved by the PTQ­(8bO2) polymer, also known as P­(Qx8O-T). The polymer presents a donor–acceptor (D–A) like structure, incorporating a thiophene ring into the Qx unit and two branches of OEG SCs with additional oxygen atoms, as shown in Figure 1a.

**Figure 1:**
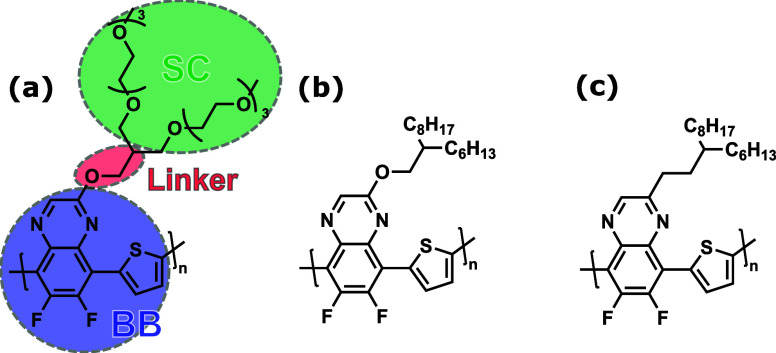
Chemical structures of (a) PTQ­(8bO2), (b) PTQ10, and (c) PTQ­(C17) polymers. The monomers are fragmented into backbone (BB), linker, and SCs units, as indicated for PTQ­(8bO2).

As the energy conversion photodynamic relies on several complex steps, the differences in performance are related to numerous variables, ranging from the packing and structure of molecules to the optical and electronic properties of molecular interfaces.^31–34^ From the structural side, significant efforts have been made to identify critical design rules for controlling local and global morphologies, for example, achieving the desirable π–π stacking with balanced phase separation domain sizes.^32,35–39^ Following an experimentally driven strategy, the combination of molecular dynamics (MD) simulations with density functional theory (DFT) and time-dependent DFT (TD-DFT) calculations has been widely employed to model the thin film formation of active layers,^32,40–44^ with a few studies dedicated to investigating the solvent’s role in the final result.^45^ Based on the structures reported by Hu et al.,^46^ chain conformations of conjugated oligomers in solution have been categorized according to the backbone arrangements, which are fundamental for interpreting spectroscopic data as they are strongly correlated with thin film morphologies.^47^

On the other hand, SCs are commonly understood as a means of increasing solubility. However, they often introduce steric and inductive effects that influence the organic semiconductors’ optoelectronic properties. Within the same family of Qx-based polymers, the branching position of SCs on the PffBT4T electron donor critically impacts the performance of fullerene-based devices, leading to PCEs that vary between 0.6% and 12%.^48–50^ These differences are related to the temperature-dependent aggregation behavior of such materials and can be controlled by finding the optimal branching position that balances both steric hindrance and aggregation effects.^51^ Toward the composition of SCs, replacing alkyl with alkoxy chains may impact electron delocalization and favor the planarity of conjugated systems.^52^ For the P7 photocatalyst, a dibenzo­[*b*,*d*]­thiophene sulfone copolymer developed by Sprick and co-workers,^53^ the additional oxygen from the alkoxy group weaken the steric repulsion between fused rings, increasing the backbone planarity compared to the aliphatic counterpart. Consequently, the optical gap is significantly reduced, the excited state lifetime is increased, and the donor character of the polymer is enhanced through the mesomeric donation from the electron-rich oxygen.^54^

In this context, to investigate the SCs’ impact on structural and electronic properties in solution, we studied PTQ­(8bO2) along with two other polymers sharing the same backbone but with different SCs, that is, PTQ10 and PTQ­(C17), presented in Figure 1b,c, respectively. PTQ10 was developed by Li et al.^55^ and combines low-cost synthesis with strong absorption, great stability, and high charge carrier mobility, serving as a model for developing new materials.^24,25^ To match the purely carbon-based SCs of nonaqueous-soluble organic semiconductors, we investigated the effect of alkoxy SCs by proposing PTQ­(C17) that replaces the oxygen from the alkoxy chain in PTQ10 with a CH_2_ group. Combining classical MD simulations, DFT calculations, and machine learning techniques with these three polymers, we vary the SC composition to assess how the polar groups affect the properties in solution at low and high concentrations and, to some extent, the properties of the solution-processed thin films. As a result, during the classical MD simulations, we identified the formation of interfaces between oligomer chains at typical π–π stacking distances for the systems solvated in chloroform and in the polar protic water/ethanol mixture. This behavior is an aggregation trace, which impacts the structural properties by reducing the oligomer’s mobility and increasing the number of intermolecular contacts. Toward the quantum description, we start by comparing different DFT and TD-DFT methods to find the optimal balance between accuracy and computational cost. Then, we determine the UV–vis spectra by employing the sequential QM/MM approach with a discrete environment description via electrostatic embedding.^56–58^ Finally, to investigate how the molecular stacking orientation impacts the absorption, we conclude the discussion with a systematic study of interface dimer models in vacuum, comparing X- and O-shaped stacking with J- and H-type aggregations.^31,59^

## Materials and Methods

### MD Simulations

In order to investigate structural properties of PTQ­(8bO2), PTQ10, and PTQ­(C17) polymers (Figure 1), classical MD simulations were performed using the GROMACS software.^60^ The polymers were described as oligomer chains with 10 monomers, and the all-atoms optimized potentials for liquid simulations (OPLS-AA)^61,62^ force field was adopted. Topology files were generated via PolyParGen software^63^ with further optimization of partial charges and torsional angles performed through DFT calculations, as described in the Supporting Information with the protocol for creating the initial oligomer structures. Initial system configurations were built by combining the Packmol code^64^ and the GROMACS toolkit. The latter was also integrated with Python scripts developed on top of several libraries, such as numpy,^65^ pandas,^66^ scipy,^67^ scikit-learn,^68^ matplotlib,^69^ and MDAnalysis,^70^ to create input files and analyze the simulation outputs. Solvent molecules were also modeled using the OPLS-AA force field parametrization developed by Jorgensen et al. for water^71^ (TIP3P), ethanol,^72^ and chloroform.^73^

For each system, two sets of simulations were performed: one with a single oligomer chain and the other with five oligomer chains. To investigate the differences between solvents, PTQ­(8bO2) was simulated in chloroform and in a water/ethanol mixture of 15:85 v/v, while PTQ10 and PTQ­(C17) were solvated only in chloroform, as they are not soluble in the aqueous solution. At first, the simulations at low concentration considered a single oligomer chain solvated at the common experimental concentration of 11.5 mg/mL used for thin film processing.^30^ After an energy minimization simulation, the system was thermalized at the *NPT* ensemble for 1 ns with a pressure (*P*) of 1 atm and a temperature (*T*) of 300 K. Then, 50 ns of *NPT* simulation was performed for the production part, also at 1 atm and 300 K. We selected 15 equally time-spaced snapshots from the trajectories at low concentrations and extracted an oligomer chain configuration from each snapshot. The configurations were separated into 3 blocks of 5 structures each and used as input for constructing 3 boxes at high concentration with 5 different oligomer chain configurations inside each box. The configurations were randomly displaced in a cubic box with different oligomers separated by at least 5 Å, to avoid introducing an initial aggregation bias, and solvated at a higher concentration of 56.1 mg/mL. Following the same protocol as the low concentration simulations, the energy of each box was minimized and thermalized before the production part, comprising a simulation at the *NPT* ensemble at 1 atm and 300 K for 500 ns. This approach was done for all four systems, that is, PTQ­(8bO2), PTQ10 and PTQ­(C17) in chloroform, and PTQ­(8bO2) in aqueous mixture, resulting in 6 μs of production divided over 12 independent simulations (4 systems with 3 simulations each).

All simulations were performed with constrained hydrogen bonds and a time step of 2 fs. Long-range electrostatic interaction was treated within the particle Mesh Ewald method (PME),^74^ while temperature and pressure couplings were described according to the modified Berendsen thermostat^75^ and barostat,^76^ respectively.

### Electronic Structure Calculations

All electronic calculations were performed at the DFT and TD-DFT levels of theory, using Gaussian16 ^77^ and ORCA^78^ computational packages. Specifically, the latter was employed for running TD-DFT calculations with double-hybrid functionals and using the Tamm–Dancoff approximation, while the former was used for all other calculations. For ground-state geometry optimization and partial charge calculations, the hybrid meta-GGA functional M06 ^79^ and the 6-311G­(d,p) Pople’s triple-ζ atomic basis set were combined with the polarizable continuum model (PCM),^80^ as detailed in Supporting Information. As discussed in the following section, a benchmark with different exchange–correlation (XC) functionals was performed, and the Coulomb-attenuating method-based functional CAM-B3LYP^81^ was selected for the TD-DFT calculations with the 6-31G­(d,p) basis set. The character of singlet excited states was further investigated through the fragment-based analysis implemented on the TheoDORE code.^82^ Multiwfn toolbox^83,84^ was also employed to compute density of state (DOS) and partial DOS (PDOS).

### QM/MM Approach

From the selected snapshots of classical MD simulations, electronic properties were determined within the sequential quantum mechanics/molecular mechanics (s-QM/MM) approach. This method selects a subset of atoms to be explicitly considered in the electronic structure calculations and another subset to be treated as point charges, preserving the system’s heterogeneity that is missing in continuum solvation models. Surrounding effects were described through an electrostatic embedding, and given the size of the oligomers, the bonds that cross the boundaries between quantum and classical regions were treated according to the H link-atom method^85^ (see Section 2 from Supporting Information for more details). The script developed for creating the input files is available on GitHub, and an additional discussion is presented in the Supporting Information.

We varied the number of atoms in the QM region to determine the optimal system size, driven by the balance between accuracy and computational cost. The number of backbone units was determined by tracking the convergence of the excited-state energies and oscillator strengths at different levels of theory. At the same time, the impact of SCs on the absorption spectra was assessed for representative configurations. From each trajectory at low concentration, 1000 equally time-spaced oligomer chain configurations were extracted to serve as input for the hierarchical clustering scheme, as implemented in clusttraj package.^86^ For each resulting cluster, the absorption spectrum of the medoid configuration with SCs explicitly included in the quantum region was compared with the spectrum treating SCs as point charges. Finally, considering the first 25 singlet excited states, the UV–vis absorption spectrum of each system was determined by averaging over 200 snapshots extracted from the MD simulations.

## Results and Discussion

### Structure and Dynamics of the Polymers in Solution

To investigate the backbone flexibility, we compared the distribution of dihedral angles that connect the quinoxaline and thiophene moieties, as shown in Figure 2. Each color matches the corresponding angle of the chemical structure on the top. Solid lines are the distributions from simulations at low concentration, while the dotted lines are the average value over the 15 oligomer chains simulated at higher concentration (3 boxes with 5 chains each), with the gray-shaded areas indicating the variation between maximum and minimum values.

**Figure 2:**
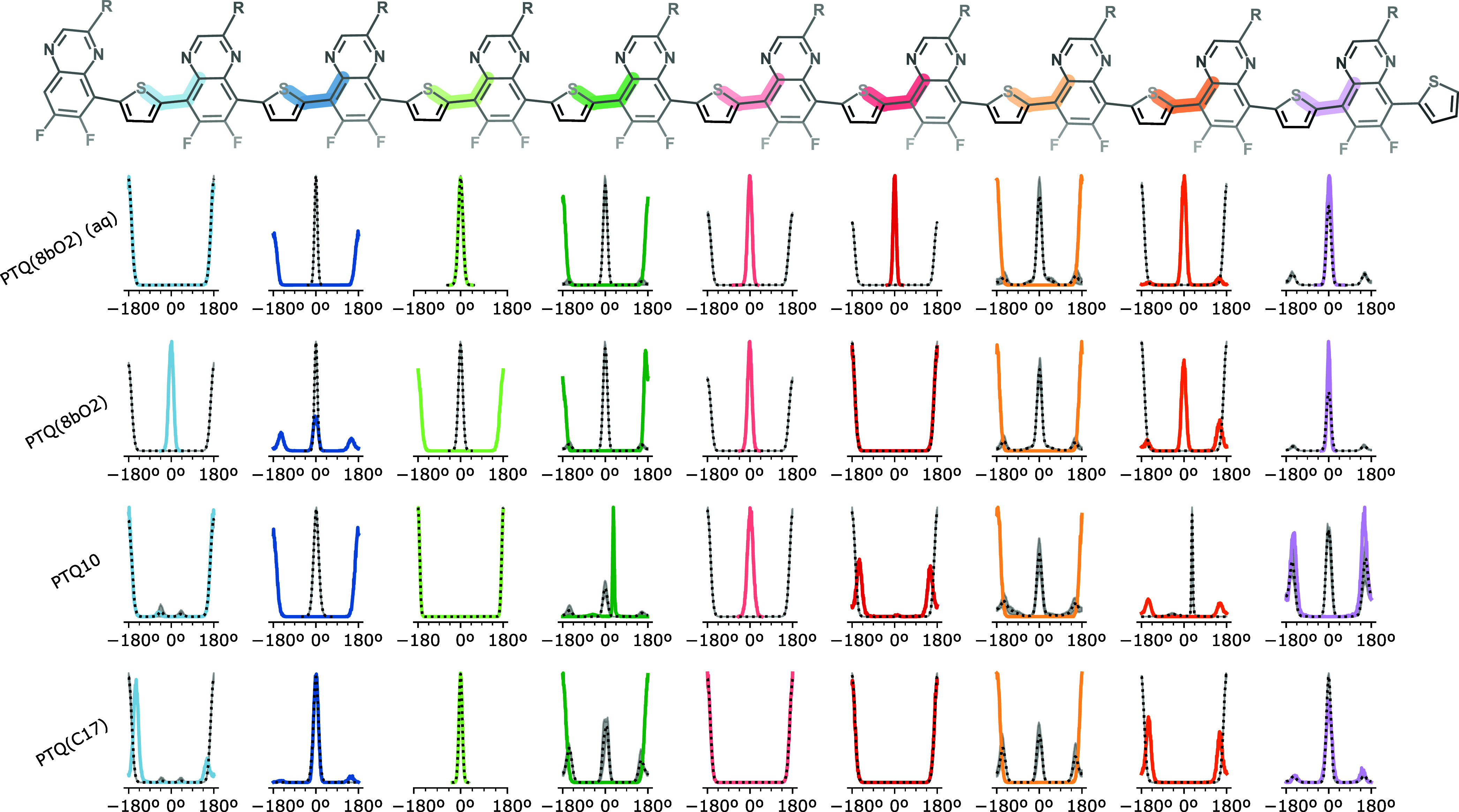
Dihedral distributions of oligomer chains at low (solid) and high (dotted) concentrations. First row corresponds to PTQ­(8bO2) in the aqueous mixture, while the other three rows are in chloroform.

Most dihedral angles are distributed around either 0 or 180°, and they converge to one of both angles depending on the input configuration since each oligomer chain has a different initial configuration. The majority of distributions have compatible values for both sets of simulations. However, for some dihedrals, such as the dark green one (forth column) of PTQ10, the single chain peak is shifted with respect to the dotted line. This shift not only reveals the effect of aggregation but also indicates a break in the conjugation length, which typically slows the intrachain charge transfer, according to the 40° tolerance threshold.^87^

Planar configurations are generally desirable, as they favor the formation of π–π stacking, but they can be an outcome of a limited simulation time scale and the force field parametrization. As shown in Figure S1, the trimer presents local energy minima around 40° to 50° with energy barriers ranging from 1 to 3 kcal/mol for the rotations, which explains the presence of more than one peak for some dihedrals and illustrates the surrounding effect over the distributions. For some of the dihedrals, the difference in energy between minimum points varies between 0.1 and 0.5 kcal/mol, suggesting similar populations for the minima around −130°, −40°, 40°, and 130° at the equilibrium. However, the increased chain size and the interaction with other molecules directly impact the distributions, changing the populations and even shifting the energy minima, as shown in Figure 2.

To compare the overall flexibility, using the initial configuration as a reference, we determined the root mean square fluctuation (RMSF) for each oligomer atom over the full time frame of all simulations, as presented in Figure 3. Since the RMSF is the time-averaged deviation of atomic positions with respect to a reference set of coordinates, higher values indicate a higher mobility of the corresponding atoms. The atom indexes were reordered to separate the polymer structures. First, we present the RMSF for the 54 atoms from the backbone of the two ends of the oligomers, followed by the other backbone atoms and finally the atoms from the SCs. As a result, we can observe that the atoms belonging to the ends of the oligomers present a consistently slightly increased mobility compared with central backbones.

**Figure 3:**
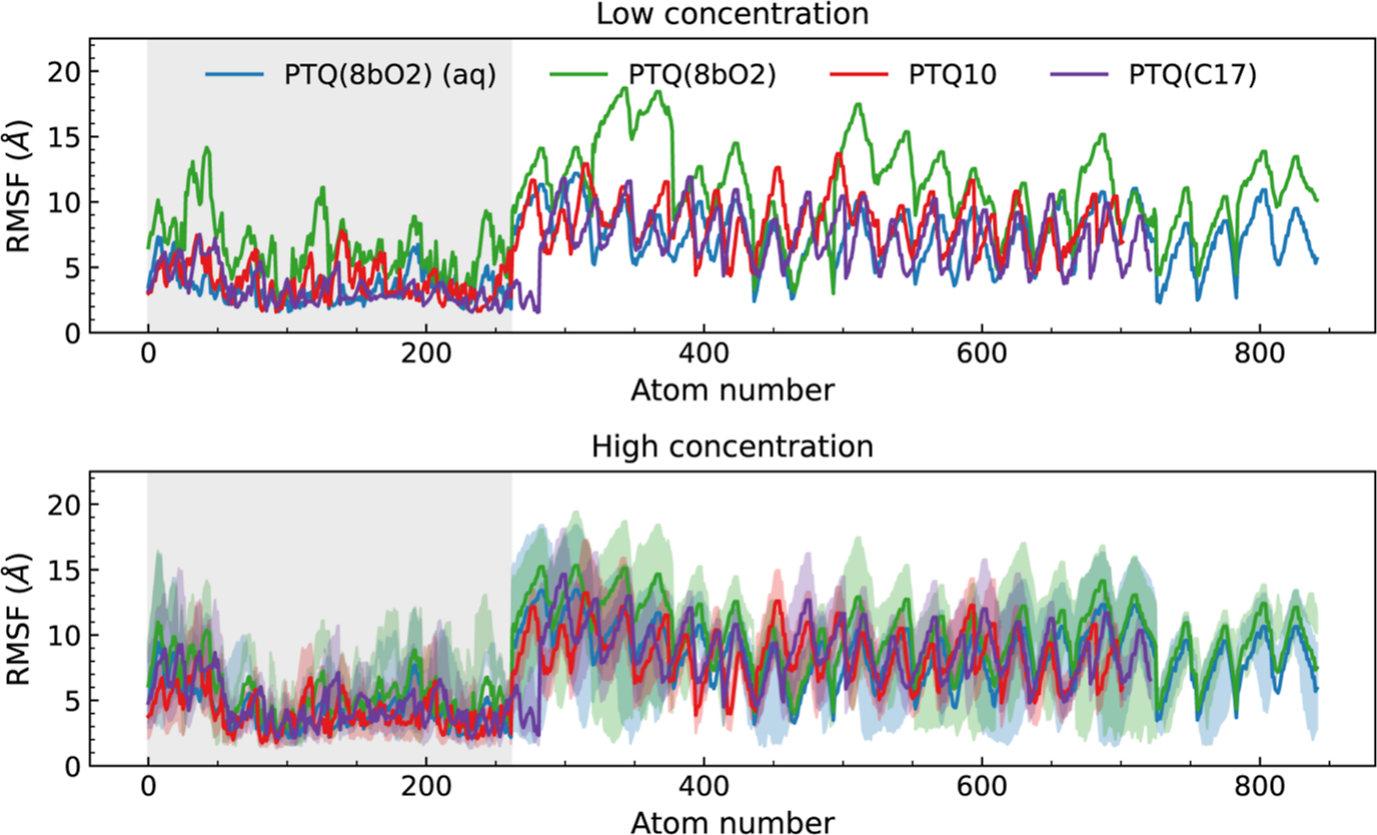
RMSF of oligomer chains at low (top) and high (bottom) concentrations. The blue curve was determined from the simulations in the water/ethanol mixture and the others from the simulations in chloroform. The gray shaded area corresponds to the backbone atoms and the nonshaded to the SCs atoms. Colored shaded areas cover from the minimum to the maximum values obtained among the 15 units.

A similar behavior is observed when comparing both panels of Figure 3, with SCs being more flexible than the rigid resonant structures of backbones regardless of the SC chemical composition. On this matter, the PTQ10 and PTQ­(C17) polymers presented the same flexibility despite replacing the oxygen in the alkoxy chains. Interestingly, the RMSF of PTQ­(8bO2) is consistently higher in chloroform than in the water/ethanol mixture, even overcoming the other lighter polymers. This result is related to chloroform’s polarity, which is high enough to be impacted by the hydrophilic behavior of OEG SCs but not too high compared to the aqueous mixture.

Also connected with flexibility, we investigated the mobility of the alkyl and alkoxy SCs. By considering the time evolution of the mean square deviation (MSD) of the furthest carbon in each branch of the SCs, we also investigated whether the SC length influences the interaction between SCs and backbones. As shown in Figure 4, dotted and dashed lines correspond to the MSD of longer and shorter SC branches of PTQ10 and PTQ­(C17), respectively, with colored shaded areas highlighting the minimum and maximum values at the high concentration.

**Figure 4:**
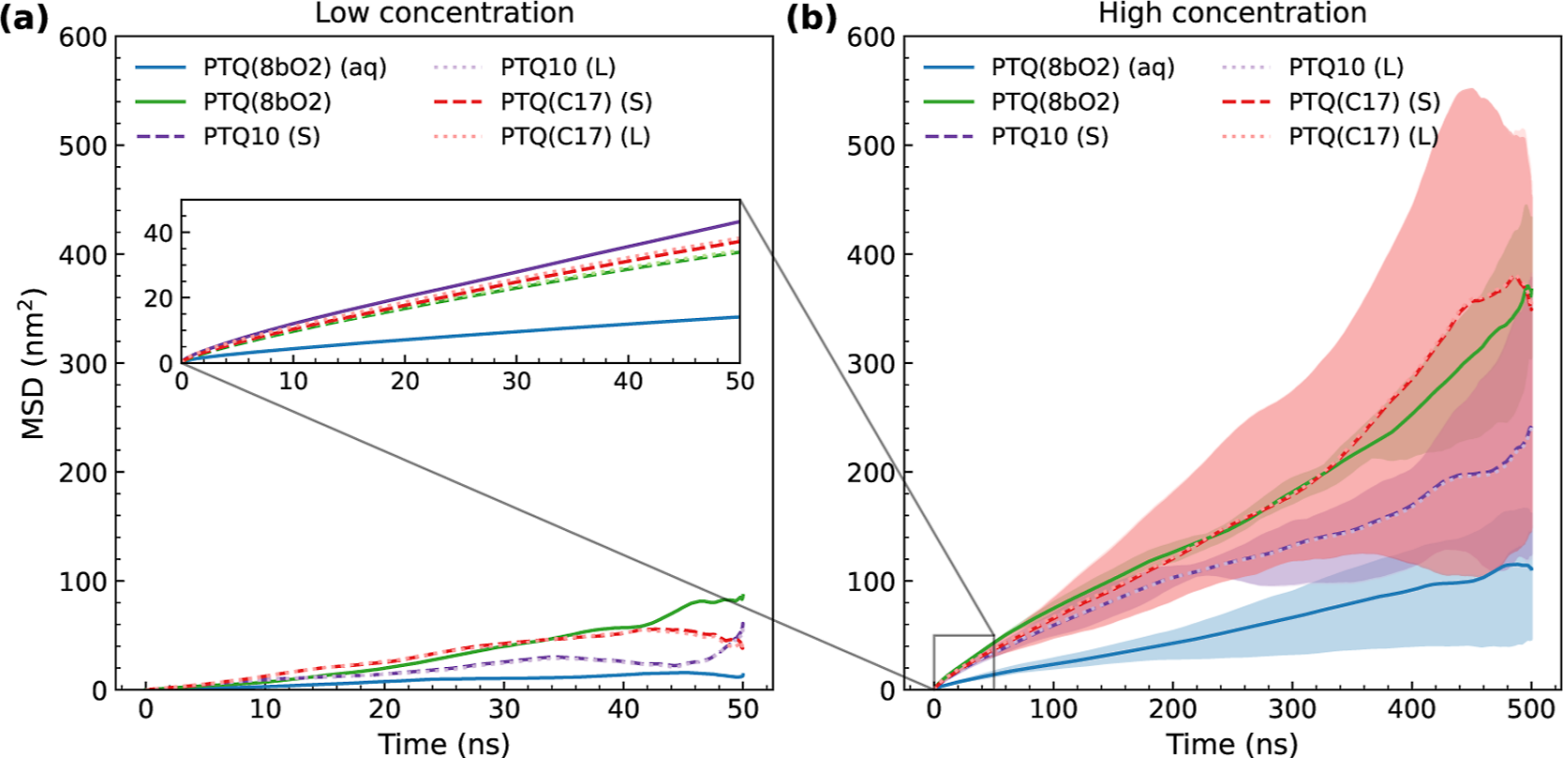
MSD of SCs as a function of time for the oligomer chains at (a) low and (b) high concentrations. The (S) and (L) symbols indicate the shorter (C_6_H_13_) and longer (C_8_H_17_) branches of the alkyl SCs, respectively.

Despite the lack of differences between longer and shorter branches, we identified solvent effects on the mobility. Oligomers in chloroform tend to present higher mobility than those in the aqueous mixture, as observed in the top panel of Figure 3 and confirmed by the converged averaged values shown in Figure 4b. From the slope of the MSD, we can estimate the diffusion coefficient, which shows a high variance when considering the 15 oligomer chains at high concentrations but is always smaller than 2.8 × 10^–6^ cm^2^/s for PTQ­(8bO2) in water/ethanol, being almost one-tenth of the self-diffusion coefficient of water molecules at room temperature.^88^ In contrast, the coefficients in chloroform for the complete oligomer chains eventually go over 6 × 10^–6^ cm^2^/s, as shown in Figure S3.

As shown in Figure 4a, oligomers at low and high concentrations have similar displacements over the first few ns of simulation. However, over time, averaged values have lower MSD due to the interaction between oligomers, indicating reduced mobility compared to the single oligomer chains at low concentration. Such interaction eventually results in aggregation, which is observed for all three polymers despite the solvent, as illustrated by the stacking between backbones of different PTQ­(8bO2) oligomers in chloroform presented in Figure S4. In other words, the solvent struggles to solubilize the polymer freely, and therefore, the system’s energy is reduced by aggregating. For most cases, the stacking starts around 100 ns and continues for the rest of the simulation. From the distance matrix, we can visually identify which atoms are close to each other and estimate the aggregation type from their relative position at the main diagonal. For example, for oligomers 2 and 3, the displacement shown in Figure S4 suggests a J-type aggregation (see Section 3.2 from Supporting Information for more information).

The reduced mobility and flexibility of PTQ­(8bO2) in the aqueous mixture can be attributed to the formation of hydrogen bonds between oxygens from SCs with the solvent molecules, especially with water. Despite the setup, the average number of hydrogen bonds between PTQ­(8bO2) and water and ethanol molecules are 49.1(47.2) and 13.9(13.6) at low­(high) concentrations, respectively, as shown in Figure S5. In fact, water molecules establish a well-defined first solvation shell, as evidenced by the radial distribution function (RDF) plots of all atoms from oligomer chains, presented in Figure S6. Even though the RDFs are similar for the different polymers in chloroform, there is a change in the ordering when shifting from single to multiple oligomer chains. The number of chloroform molecules close to PTQ­(8bO2) at short distances becomes higher than those of the other two polymers, suggesting a reduced aggregation in the presence of polar SCs. This results from the stronger interaction between oligomers with nonpolar SCs, reflected in the higher number of intermolecular contacts presented in Figure 5.

**Figure 5:**
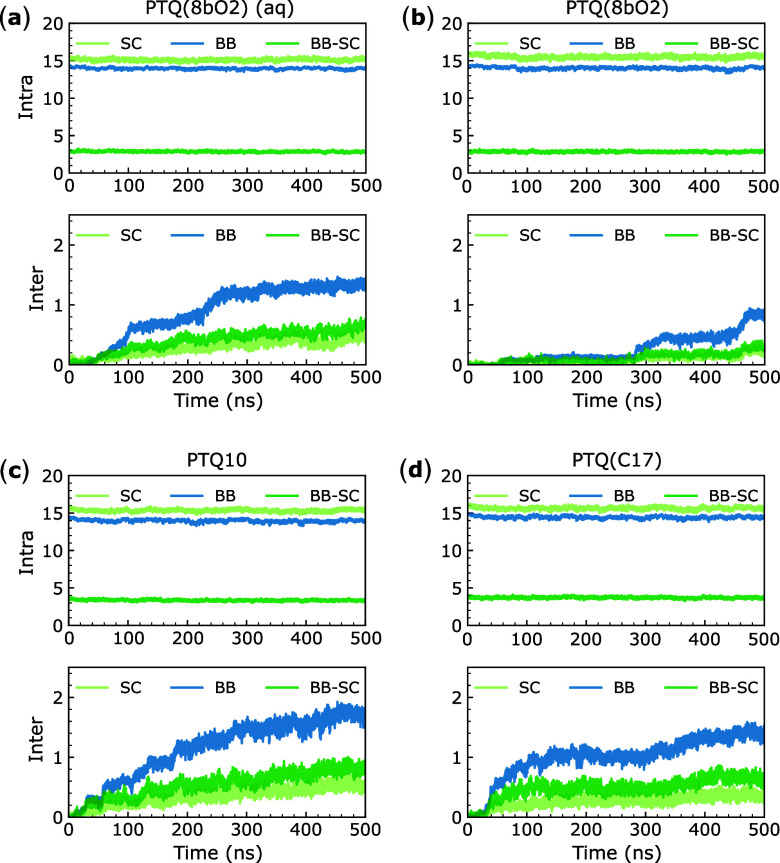
Average number of intra (top panel) and intermolecular (lower panel) contacts per atom for (a) PTQ­(8bO2) in water/ethanol 15:85 v/v mixture, (b) PTQ­(8bO2), (c) PTQ10, and (d) PTQ­(C17) in chloroform. BB, SC, and BB-SC stand for contacts between backbone units, SC units, and backbone and SC units, respectively.

A contact corresponds to a pair of atoms that belong to different fragments and are closer to a given threshold. The common value for all-atom simulations of 4.5 Å was adopted,^41^ and, for each system, we tracked the time evolution of the average number of inter and intramolecular contacts per atom (see Figure S7 for absolute contacts). As shown in the top panels of Figure 5 and center panels of Figure S7, the number of intramolecular contacts remains constant throughout the simulation. Such behavior indicates that the oligomer chains remain similar to the initial configurations, that is, the oligomers do not curl, preserving the rod-like conformations.^46^

The differences in the average number of intramolecular contacts are related to the polymer’s structure and composition. As illustrated by the PTQ­(8bO2) snapshot in Figure S8, there is a high number of intramolecular contacts between atoms belonging to the backbone (BB) or to the SCs, which results in the average value per atom around 15, as shown in Figure 5. Nevertheless, the larger distances between backbones and SCs result in a smaller average value for intramolecular contacts between SCs and backbone atoms (BB-SC). On the other hand, the number of intermolecular contacts increases during the simulation of all systems. Because we constructed the boxes ensuring at least a 5 Å distance between atoms from different oligomer chains and thermalized the system for 1 ns before production, the intermolecular contacts start close to zero and increase due to the interaction between oligomers. When comparing the four systems, PTQ­(8bO2) in chloroform is the least aggregated, consistent with the higher RDF values shown in Figure S6 and the UV–vis spectra presented in the following section. The lower diffusion coefficient of PTQ­(8bO2) oligomer chains in aqueous mixture also supports the higher aggregation (Figure S3). As the number of intramolecular contacts remains constant and the number of intermolecular contacts between backbone units is higher than that between SCs, it indicates that the backbones get closer to each other. Such behavior can be visually identified, as shown in Figure S4a,b, and is in agreement with the π–π stacking experimentally observed in thin films of organic semiconductors.^9,35,89^

### Validation of Backbone Size and Exchange–Correlation Functional

Given the electronic structure’s sensitivity to the level of theory and system size, we conducted a benchmark to determine the optimal number of monomers and the most suitable DFT exchange–correlation functional. As we are interested in the absorption spectrum, the energy and oscillator strength of the first bright state were determined using B3LYP,^90^ M06,^79^ CAM-B3LYP,^81^ LC-ωHPBE^91^, and RI-SOS-PBE-QIDH^92^ for 1 up to 6 monomers. The 6-31G­(d,p) atomic basis set was employed for the former 4 XC functionals, while the def2-SVP set was used for the latter. The double-hybrid functionals, such as PBE-QIDH, provide accurate description of excited states,^93–95^ but we only used it as reference due to the significant increase in the computational cost. Here, we included the linker groups (−CH_2_CH_2_CH or –OCH_2_CH) in the QM region and saturated the single bonds with hydrogen atoms, as previously described. For each system, the geometry was optimized for the isolated molecule at the M06/6-311G­(d,p) level of theory prior to the TD-DFT calculations. The brightest state was always the first excited state (S_1_), whose energy and oscillator strength are presented in Figure 6. Results obtained with LC-ωHPBE are divided into two parts, that is, OT-SRSH and OT-SRSH-PCM, according to the tuning procedure. The former only performs calculations in vacuum, while the latter describes the solvent interaction at the continuum level via PCM. These approaches were developed by Kronik et al.^96–98^ and include optimizing the three parameters of the Coulomb-attenuating partitioning method of the electrostatic interaction.

**Figure 6:**
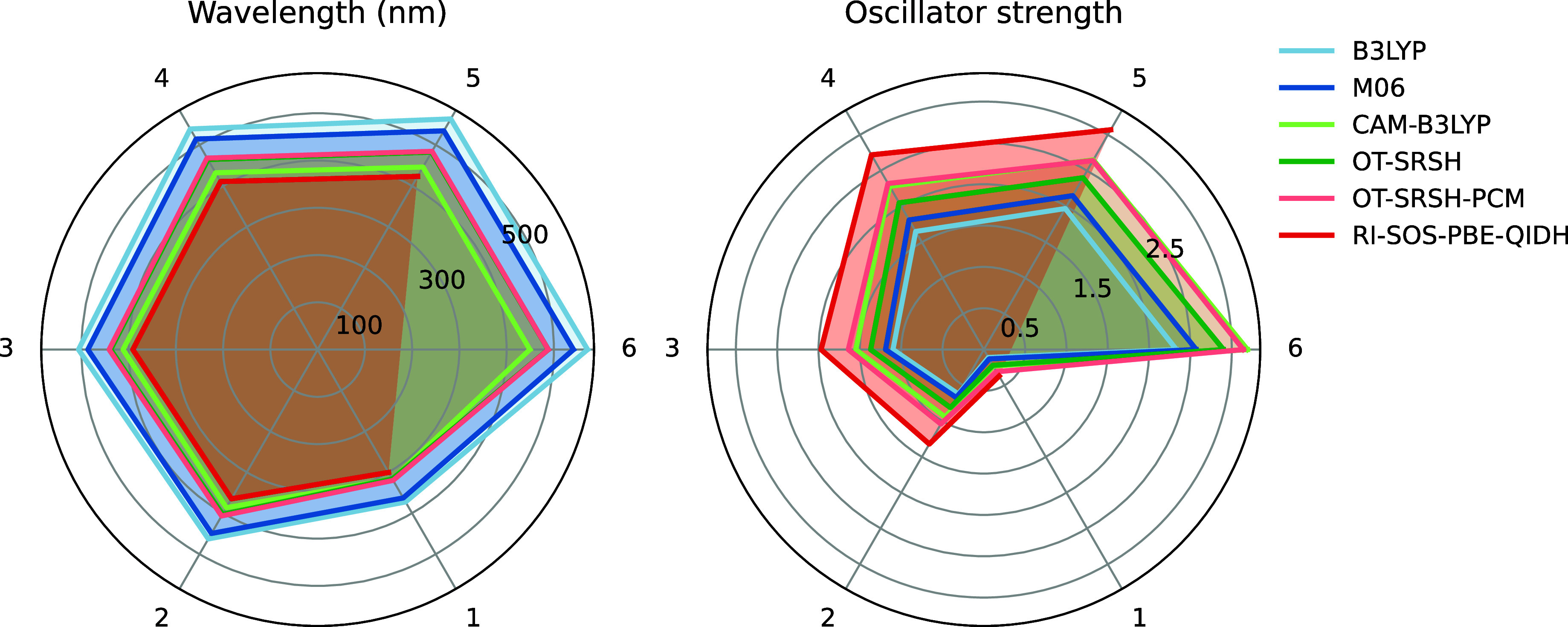
Excitation energy and oscillator strength of the first excited state (S_1_) as a function of the number of backbone units (without SCs) for different levels of theory. The def2-SVP atomic basis set was employed for RI-SOS-PBE-QIDH calculations and the 6-311+G­(d,p) for the all other XC functionals. All geometries were optimized at the M06/6-311G­(d,p) level of theory.

When comparing XC functionals, the long-range correction produces higher excitation energies and oscillator strengths. These differences have also been previously reported for other systems, typically blue-shifting the spectrum, which, in our case, resulted in a good agreement between LC-ωHPBE (OT-SRSH-PCM) and CAM-B3LYP with the RI-SOS-PBE-QIDH reference. Despite the contrast compared to the well-established B3LYP, this behavior is consistent, showing better performance as we climb the Jacob ladder of XC functionals.^99^ Since the differences between both methods are minor, we selected the standard CAM-B3LYP for computing excited state properties.

Concerning the system size, the excitation energies consistently decrease as we increase the number of monomers. However, the change gradually decreases, showing a convergence for all functionals (Table S1). For example, with CAM-B3LYP, the energy of S_1_ reduces 0.7 eV when moving from 1 to 2 monomers but only 0.02 eV from 5 to 6 backbones, resulting in a redshift of the absorption spectrum of 69.2 and 3.2 nm, respectively. Therefore, we consider five as the ideal number of monomers for quantum calculations.

### Impact of SCs over Excited-State Properties

The role of SCs in electronic excitation is generally secondary. As previously discussed, they are added to improve the solubility of materials and mainly influence their morphological properties. To investigate their impact on the UV–vis absorption spectrum, we selected representative configurations to compare the results obtained with SCs explicitly included in the QM region to those treated as point charges. In contrast to considering optimized geometries, this approach allows us to account for the system flexibility, especially SCs, which could eventually get tangled up or even avoid π–π stacking by laying on the backbone units. We started by selecting 1000 equally time-spaced snapshots from the MD simulations at a low concentration. Then, from each snapshot, we extracted the oligomer chain structures and used them as input for the hierarchical clustering scheme. We adopted the average linkage method for merging the clusters, as it was previously used to cluster configurations extracted from MD simulations owing to the good balance between the number of clusters and their similarity.^100^ Combining the silhouette score^101^ with visual analysis of the dendrograms (Figure S9), we obtained a balanced description by considering the RMSD threshold of 8 Å, which resulted in 3 clusters for PTQ10, PTQ­(C17), and PTQ­(8bO2) in the aqueous mixture and 11 clusters for PTQ­(8bO2) in chloroform. For each cluster’s medoid, we computed the first 25 singlet excited states of the five central monomers to determine the absorption spectra shown in Figure 7.

**Figure 7:**
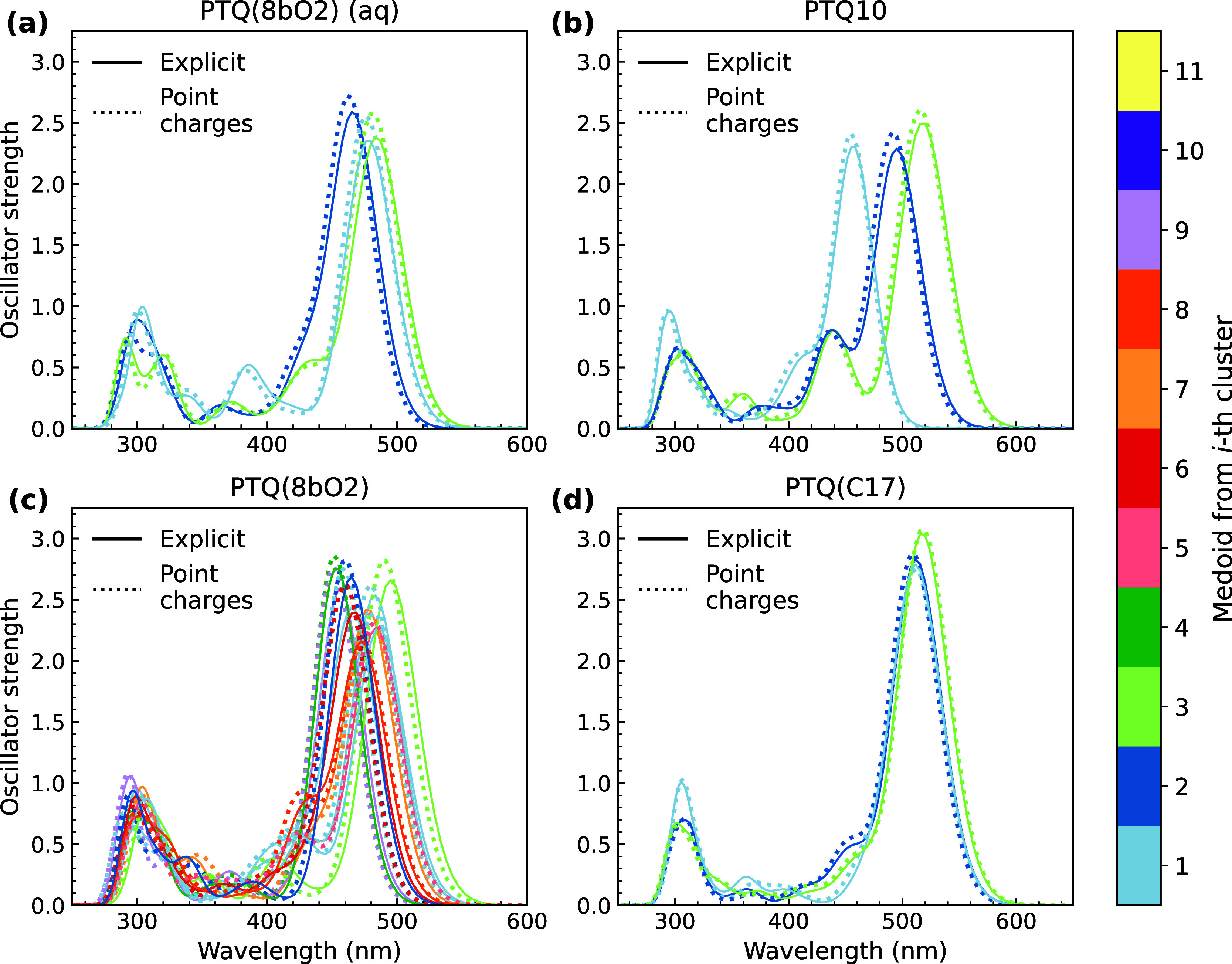
Absorption spectrum of the medoids from each cluster of (a) PTQ­(8bO2) in water/ethanol mixture and (b) PTQ10, (c) PTQ­(8bO2), and (d) PTQ­(C17) in chloroform. Calculations were performed at the CAM-B3LYP/6-31G­(d,p) level of theory, considering the 5 central monomers.

The comparison between dotted and solid lines reveals that the electrostatic interaction with point charges can accurately reproduce the SC effect over the first absorption peak at lower energies. For PTQ10 and PTQ­(C17), even the second absorption band shows no difference between the approaches. The resonant structures in the backbone contribute to reinforcing the π-shaped nature of orbitals close in energy to the HOMO and LUMO, preserving the typical π–π character of excited states’ dominant transitions. Changes in the band shape start to appear in the presence of polar SCs and should impact excited states below 300 nm. Adding oxygen atoms to the SCs contributes to delocalizing molecular orbitals close to the energy gap toward the SCs, as evidenced by the DOS presented in Figure 8. While the DOS was determined from the convolution of Gaussian functions centered in the MO energies, the partial DOS projects the orbital into the atomic basis functions at specific atoms. Therefore, by selecting a collection of desired atoms, we can get the contribution of a given fragment and combine it for all orbitals in an energy window. In particular, the plots in Figure 8 show the medoids’ total DOS and PDOS projected into the backbones (BBs), SCs and linker groups, for each system’s most populated clusters. Specifically, clusters 3, 1, 3, and 4 have 90%, 92%, 76%, and 21% of the configurations of PTQ10, PTQ­(C17), and PTQ­(8bO2) in the aqueous mixture and PTQ­(8bO2) in chloroform, respectively. By comparing the left and right plots of Figure 8, for PTQ10 and PTQ­(C17), all orbitals from −8 eV up to 1 eV are delocalized exclusively over the backbone. On the other hand, for PTQ­(8bO2), the MO delocalization over the SCs is observed for energies higher than −7 eV. Therefore, one should expect polar SCs to be directly involved in absorbing photons in the middle and far ultraviolet ranges. The fragment-based analysis supports this statement by capturing significant charge-transfer (CT) transitions from the SCs toward the backbone only for the PTQ­(8bO2) system, as shown in Figure S10. This approach projects the natural transition orbitals of a given excitation over the atomic basis functions centered at specific atoms,^82,102^ providing information concerning the electron and hole delocalization over molecular fragments. Additionally, we observed that the excitation character is highly dependent on the XC functional employed. Exchange–correlation functionals without long-range correction, such as B3LYP and M06, tend to underestimate the overall excitation energy and overestimate the CT character of singlet excited states, while CAM-B3LYP reproduces the results obtained with the double-hybrid functional. For further details, see Section 5.2 in Supporting Information.

**Figure 8:**
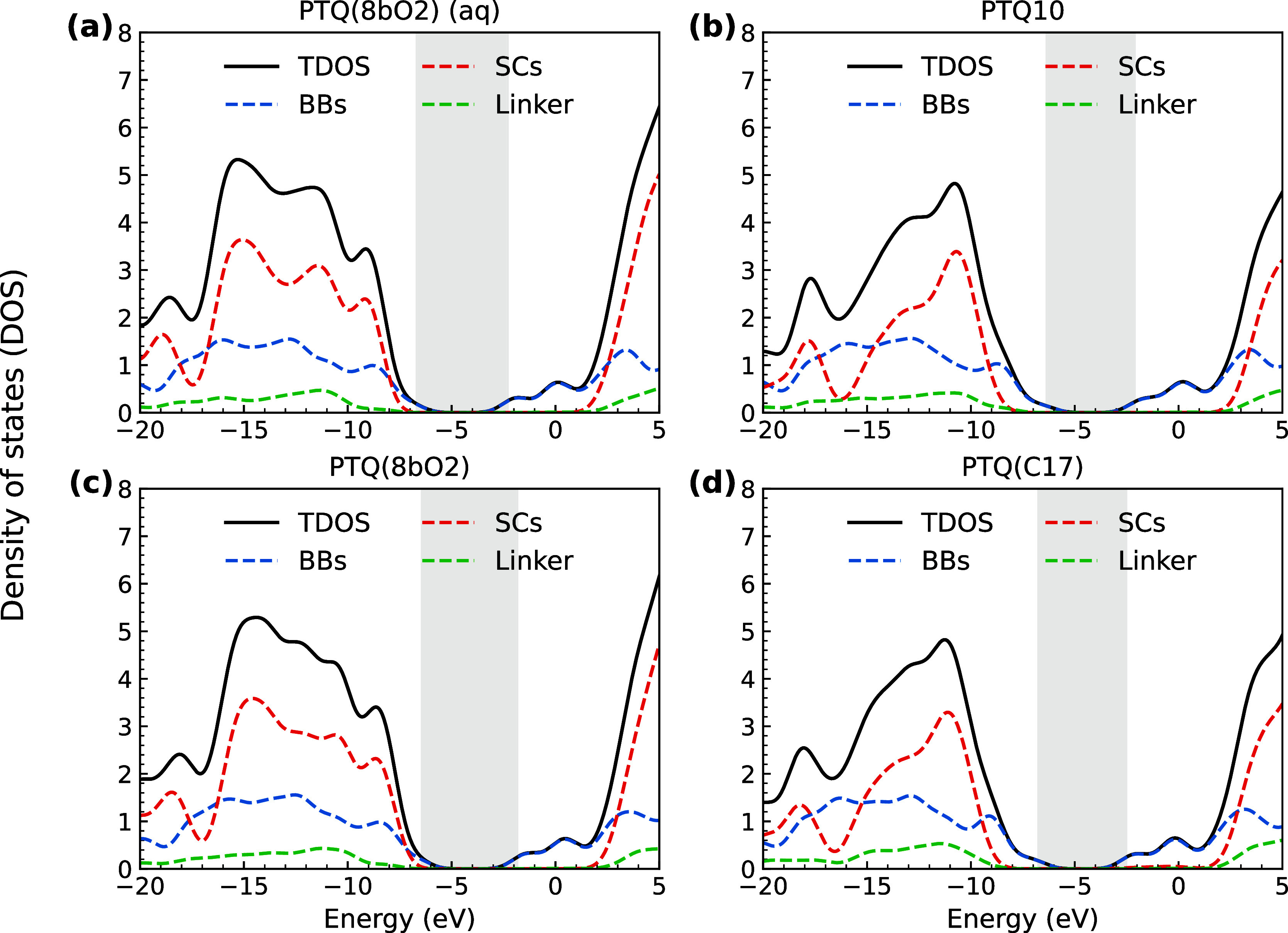
Partial and total DOSs for the most populated clusters of each polymer in (a) aqueous medium and (b–d) chloroform. Calculations were performed at the CAM-B3LYP/6-31G­(d,p) level of theory, considering the 5 central monomers with SCs as the QM region, while the other atoms were treated as point charges.

When comparing the contribution of linker groups, formed by the atoms close to the backbone (Figure 1), it indicates that the change from alkyl to alkoxy has a negligible impact on the orbital spatial distribution. Nevertheless, it seems to influence the energy range of the first absorption peaks shown in Figure 7. PTQ10 and PTQ­(8bO2) peaks present a higher energetic disorder of S_1_, suggesting that oligomer chain conformations in solution are less diverse in the presence of purely aliphatic SCs.

### UV–Vis Absorption Spectra

With the level of theory established, we determined the UV–vis absorption spectra via the s-QM/MM method. From each simulation at low concentration, we extracted 200 equally time-spaced snapshots to get a sample of configurations and considered five central monomers without SC branches in the QM region. Following the same approach employed in the methodology validation, the connection between quantum and classical regions was established by saturating the single bonds of SC branches and adjacent monomers with hydrogen atoms, as proposed by the H link-atom approach. All other atoms up to a minimum distance of 30 nm were included in the classical region and treated as point charges during the QM calculations, as illustrated by Figure S2.

Since aggregation is expected to impact the shape and range of the absorption profile, we also considered 200 equally time-spaced configurations containing explicitly π–π stacked oligomers of PTQ­(8bO2) in the aqueous mixture. In this case, the QM region was formed by 10 monomers without SCs arranged as five backbones belonging to one oligomer chain on top of the other five from a different chain. We also measured the absorption spectra of PTQ­(8bO2) and PTQ10 at low concentrations of 0.03 mg/mL, and we compared them with the calculations, as shown in Figure 9. Details on the experimental procedure can be found in Section 6 from Supporting Information.

**Figure 9:**
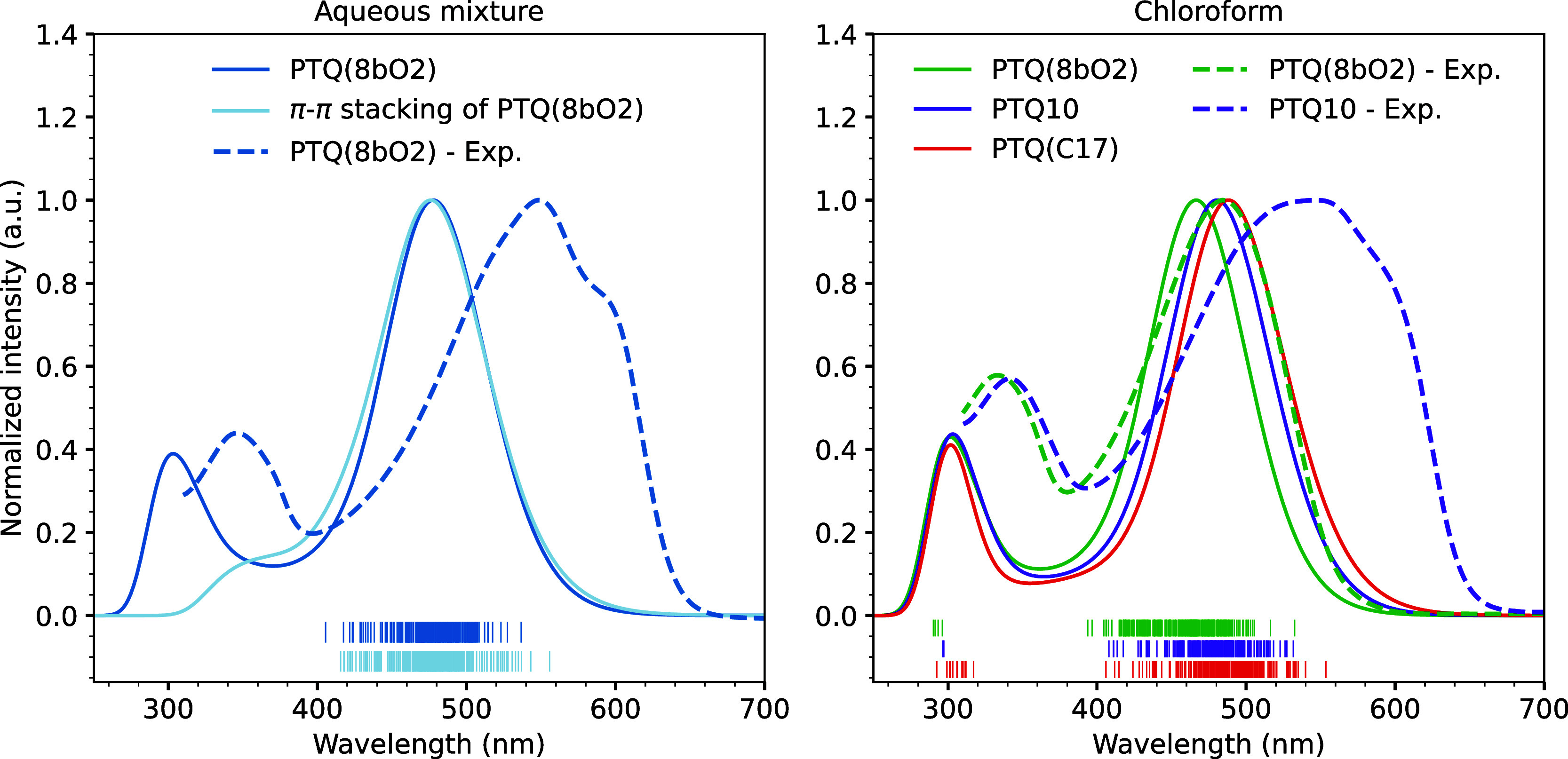
Theoretical and experimental absorption spectra of PTQ10, PTQ­(C17), and PTQ­(8bO2) in the aqueous mixture and in chloroform. Experimental results were measured at the concentration of 0.03 mg/mL and at room temperature, and each theoretical spectrum was determined via the s-QM/MM approach with 200 configurations. π–π stacking configurations were extracted from the high-concentration simulations and all the other configurations from the low-concentration simulations. Vertical lines correspond to the excitation energies of states with oscillator strength higher than 0.2 (after normalization).

When comparing the effect of SCs over the theoretical spectrum in chloroform, we observe that the first absorption peak of the PTQ­(C17) system is slightly red-shifted compared to that of PTQ10, which is red-shifted with respect to PTQ­(8bO2). Specifically, the brightest peaks are centered at 488, 480, and 466 nm for PTQ­(C17), PTQ10, and PTQ­(8bO2), respectively. This gradual shift of the spectrum results from the increased system polarity that stabilizes charge transfer states, increasing the CT character of the low-energy excited states. Even though the SCs are not explicitly included in the QM region, the electrostatic embedding captures the polarization differences from one SC to another. By comparing the experimental results for PTQ­(8bO2) in both solvents, the red shift of around 100 nm of the low-lying absorption band suggests that the polymer is showing aggregation signs in the aqueous mixture. Moreover, the shoulder-like structure around 600 nm correlates with the features usually observed for polymeric thin films.^103^ From temperature-dependent absorption measurements of conjugated polymers, one can identify that the spectrum in solution at low temperatures is comparable to the results for thin films. For example, the first peak of the PffBT4T-2OD electron donor polymer shifts around 150 nm when the temperature increases from 25 to 85 °C, suppressing the double peak structure observed in the thin film spectrum.^104^ Likewise, we can identify aggregation in PTQ10 given the red-shifted purple dotted curve in the right panel of Figure 9, which has the broad and irregular brightest peak.

Regarding the TD-DFT calculations on stacking aggregates, one should expect that the π–π interactions would contribute to delocalizing the low-energy excitations over both oligomer’s backbones, stabilizing the excitation energies. However, aside from the higher number of excited states at low energies (due to the larger system size), which reduces the energy range coverage of the first 25 excited states, the spectrum of the stacked system is equivalent to that considering a single oligomer chain. Therefore, aggregation’s effects are beyond the proposed model, encompassing only two oligomer chains with five monomers each. Even though our model includes molecular interfaces, this limitation should not depend on the atomic basis set given the negligible differences when shifting from double- to triple-ζ or adding diffuse functions, as shown in Figure S12. The balance between short- and long-range exact HF contributions in the XC functional could impact the results, as the optimal long-range parameter directly depends on the system size,^105,106^ but the size itself plays a critical role. Considering that the experimental number-averaged molecular weights go up to 47 kg/mol^–1^ (see Section 6 from Supporting Information), one should expect more than 50 monomers per oligomer chain, which will form aggregates in the μm scale. Since modeling such system sizes is unfeasible even for all-atom MD, reproducing the absorption of aggregates requires alternative approaches.

We also determined the spectrum of π–π stacking configurations by using the polarizable continuum model (PCM) to account for the environment. Similarly, we employed the hierarchical clustering technique with the 200 configurations and ran the calculations for the medoid from each of the five resulting clusters (Figure S9e). To assess the impact of the dielectric constant on the results, we determined the spectrum on chloroform and on the aqueous mixture. For the solvent-related parameters of the mixture, we considered the values for ethanol along with the effective static dielectric constant determined according to Bruggemann’s equation, considering the 15:85 v/v proportion of water/ethanol used for the thin film production (ϵ = 30.10). Given the small difference between water and ethanol refractive indices (i.e., 1.334 ^107^ and 1.365,^108^ respectively), the optical dielectric constant of ethanol was adopted. In summary, changing the solvent had a minor impact on the spectrum, shifting around 0.03 eV (<5 nm), as shown in Figure S13. Nevertheless, the continuum model had a better agreement with the aggregated experimental results, shifting the first absorption peak from 470 to around 500 nm, depending on the stacking configuration.

From the MD simulations, we observed the stacking of oligomer chains for all systems, generally combining different types of aggregation. As we do not have a preferential type of stacking, when considering a set of 200 configurations, the averaged spectrum misses traces that separate from one aggregation type to another. Therefore, to investigate how the arrangement impacts absorption, we constructed idealized interfaces of H- and J-type aggregations as well as O- and X-shaped stacking dimers. To minimize the medium effect, we determined the excited states in a vacuum and compared the interface absorption spectrum with the spectrum of the isolated oligomer chains. Given the large size of the systems, we considered three monomers without SCs per oligomer. The interfaces were built by duplicating the optimized geometry of three bonded monomers (Section 1 from Supporting Information) and stacking according to the type of packing, as shown in Figure 10.

**Figure 10:**
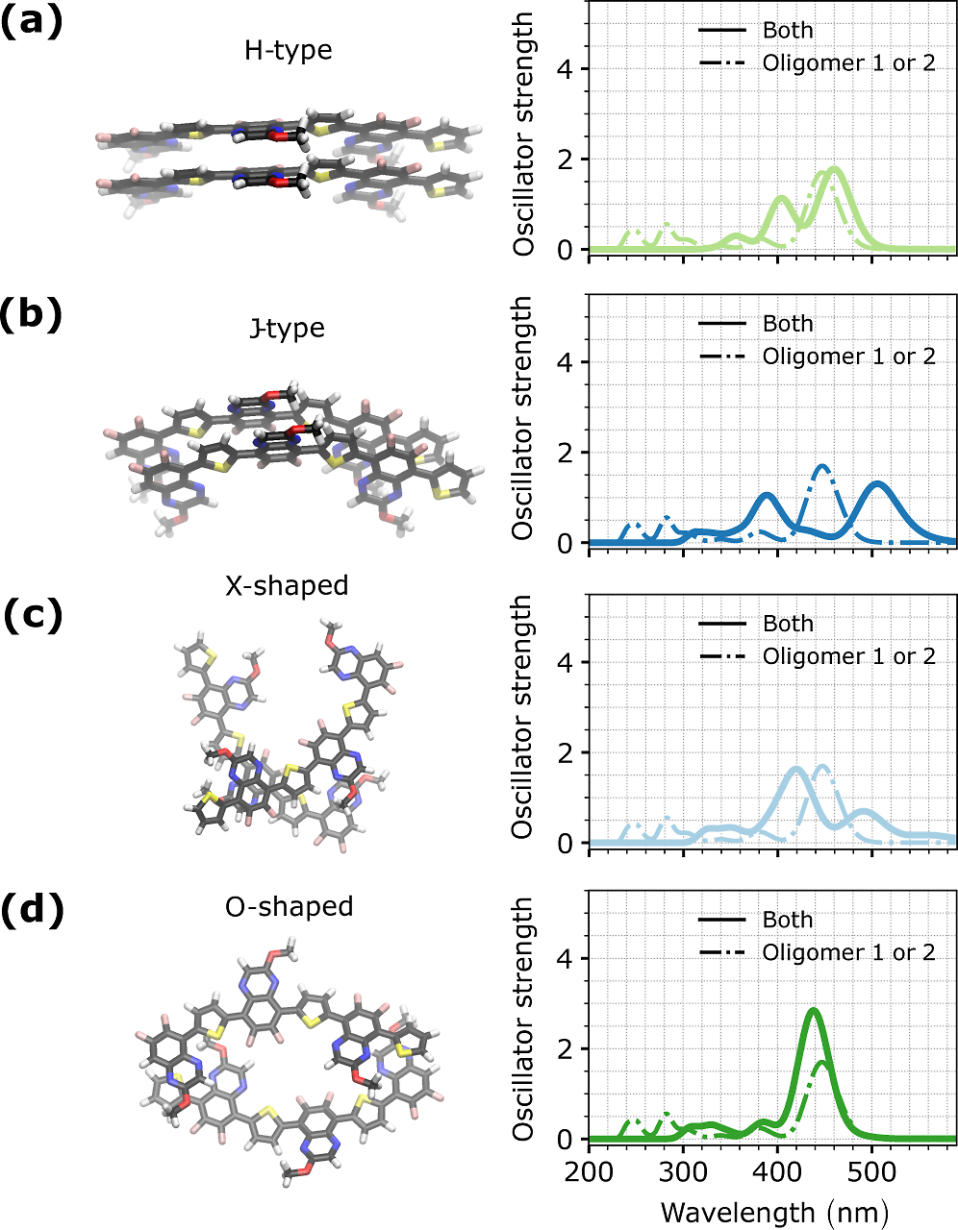
Absorption spectra of different idealized (a) H-type, (b) J-type, (c) X-shaped and (d) O-shaped stacking configurations of PTQ-based interfaces in vacuum. The system spectrum (solid lines) is compared with the spectrum of each isolated oligomer for idealized interfaces. Calculations were performed at the CAM-B3LYP/6-31G­(d,p) level of theory considering 3 monomers per oligomer chain without SCs, as shown by the configurations at the right of each plot.

In contrast to the spectra presented in Figure 9, considering a single idealized configuration without an environment description exposed the differences between the single and dimer spectra. Additionally, the dimer spectrum was sensitive to the molecular orientation, presenting a double peak structure for all cases except the O-shaped dimer. According to Kasha’s rule,^31^ the different signs of the Coulomb coupling in J- and H-type aggregations should shift the dimer spectrum to red and blue with respect to the oligomer spectrum, respectively. However, the break in planarity and the combination of electron-withdrawing and electron-donating groups that form the backbone of such molecules result in a mixed description of the excitations. The presence of donor and acceptor groups introduces polarization and asymmetry in the electronic structure, creating partially delocalized and CT excited states that directly impact the absorption spectra.^89,106^ As shown in Figure 10, the deviation from the oligomer spectrum is more pronounced for the J- and H-type dimers, which have a stronger π–π interaction when compared to that of the X- and O-shaped dimers. Finally, despite the potential contribution from the vibrational motion, which can increase the vibronic couplings, impacting the absorption profile and the intermolecular charge delocalization,^31^ such effects tend to be mitigated by the reduction of the system’s degrees of freedom after the thin film formation.

## Conclusions

Aqueous-processable electron donor quinoxaline-based polymers were studied via classical MD simulations and (TD)-DFT calculations. By considering three systems, that is, PTQ­(8bO2), PTQ10, and PTQ­(C17), we investigated the impact of SCs on structural and optical properties in solution. We tracked the aggregation evolution by considering the polymers in infinite dilution and a high-concentration setup. Due to the presence of atoms with high electronegativity, the system evolution was driven by the thermodynamical competition between the stacking of solute resonant structures and the formation of hydrogen bonds between solute and solvent molecules. Combining the higher flexibility revealed by the RMSF of SC atoms with the number of intermolecular contacts between oligomer chains, we identified that PTQ­(8bO2) in chloroform has a solubility higher than those of the other systems. Nevertheless, we observed the stacking of oligomer chains for all systems, with a significantly lower mobility of PTQ­(8bO2) in a water/ethanol 15:85 v/v mixture. Such a strong interaction was supported by the experimental absorption spectra, presenting traces of aggregation for PTQ10 in chloroform and PTQ­(8bO2) in the aqueous mixture.

Regarding optical properties, the theoretical UV–vis absorption spectrum is sensitive to the system size and level of theory. Increasing the number of backbone units reduces the excitation energies, which converge well when considering five monomers in the QM region. The addition of long-range correction in the XC functionals also had a clear effect on the spectrum, increasing the excitation energies and improving the agreement with the experimental data of the nonaggregated PTQ­(8bO2) spectrum in chloroform. From the PDOS plots, one could identify the effect of polar groups, contributing to delocalizing molecular orbitals closer to the energy gap over the SCs. As a result, for a specific set of configurations, we observed the direct contribution of SCs to the excitation character of excited states with lower energy.

Despite the size limitation to model the aggregation effects over the UV–vis absorption spectrum, the electrostatic embedding within the s-QM/MM approach accurately described the brightest absorption peak of nonaggregated systems. Finally, the idealized interface models presented individual profiles in the spectrum, enabling the identification of different types of stacking. However, the presence of donor and acceptor moieties introduces charge-transfer excited states that deviate the spectra from Kasha’s rule, showing mixed signatures from different standard aggregation types.

As a result, our findings contribute to understanding the behavior of energy-harvesting polymers in solution, suggesting a strong interaction between oligomeric chains that results in aggregation even before the thin films. Despite the need for refinements to model the optical properties of aggregated systems, we presented a methodological approach that can reproduce the experimental trends. It is suitable for low concentrations, extensible to other systems, and can eventually be combined with coarse-grain force fields to enable the simulation of larger system sizes for longer time scales. Enhanced MD sampling techniques could also be an alternative to improve conformational sampling, further contributing to the development of new materials.

## Supplementary Material



## Data Availability

Scripts developed for creating input files and analyzing the results are freely available on GitHub. The data are available upon request.

## Notes

Energy profile of torsional angles, H-link method, diffusion coefficients, distance matrix plots, hydrogen bond analysis, radial distribution functions, absolute number of contacts, dendrograms from hierarchical clustering, benchmark of excitation energies and oscillator strengths, fragment-based analysis of excited states, benchmark of atomic basis sets for UV–vis absorption spectra, PCM absorption spectra, instrumental and measurements (PDF)
