# Supplementary material for: Assessing Structural and Optical Properties of PTQ10-Based Donor Polymers in Solution for Eco-Friendly Photovoltaics: A Multiscale Modeling Study
Source: J Phys Chem B. 2025 May 30;129(23):5887–900. doi: 10.1021/acs.jpcb.5c01972 (PMC12169692; doi:10.1021/acs.jpcb.5c01972)
Supplement: Supplementary file 1 [file jp5c01972_si_001.pdf]

Supporting Information

Assessing Structural and Optical Properties of PTQ10-based Donor Polymers in Solution for Eco-Friendly Photovoltaics: A Multiscale Modeling Study

Rafael B. Ribeiro[1,2,3], Leandro R. Franco[3,4],
Alexandre Holmes[4], Tárcius N. Ramos[5], Ergang Wang[4,*]
Márcio T. do N. Varella[1,*] and C. Moyses Araujo[2,3,*]

[1] *Institute of Physics, University of São Paulo, Rua do Matão 1731, 05508-090 São Paulo, São Paulo, Brazil*

[2] *Materials Theory Division, Department of Physics and Astronomy, Uppsala University, 75120 Uppsala, Sweden*

[3] *Department of Engineering and Physics, Karlstad University, 65188 Karlstad, Sweden*

[4] *Department of Chemistry and Chemical Engineering, Chalmers University of Technology, Göteborg SE-412 96, Sweden*

[5] *Theoretical Chemistry Lab, Unit of Theoretical and Structural Physical Chemistry, Namur Institute of Structured Matter, University of Namur, Rue de Bruxelles, 61, B-5000 Namur, Belgium*

*✉: ergang@chalmers.se

*✉: mvarella@if.usp.br

*✉: moyses.araujo@kau.se

Contents

1 Oligomer structures and force field parameterization **S5**

S1 Energy profile of dihedrals fitted from rigid scan. (a) 2D representation of the trimer with fitted dihedrals highlighted. (b-e) Total (top panel) and torsional (lower panel) energies as a function of the dihedral angle. Each plot respectively corresponds to the energy profiles of the angles highlighted in (a), from left to right. S6

2 H link-atom method **S7**

S2 Representative configuration of a quantum region (vdW spheres) defined as 5 monomer backbones with all other atoms closer than 30 nm described as partial charges. This configuration was extracted from the simulation of a single oligomer chain of PTQ(8bO2) in chloroform. S7

3 MD simulations **S9**

3.1 Diffusion coefficients S9

S3 Time evolution of diffusion coefficients from single oligomer simulations (left) and averaged over the 15 oligomers from the 3 boxes with 5 oligomers each (right). Vertical bins in the right plot correspond to the variation between maximum and minimum values. S9

3.2 Distance matrix plot S10

S4 Aggregation of a representative snapshot from one of the simulations of 5 PTQ(8bO2) oligomer chains in chloroform. Snapshot of the (a) complete system and (b) only showing the resonant structures from backbones. Distance matrix plots (c) between all oligomer atoms and (d-e) between atoms from oligomers 2 and 3 that are aggregated. S11

3.3 Hydrogen bonds S12

S5 Hydrogen bonds of PTQ(8bO2) in water:ethanol mixture. Distribution of number of bonds (left), distances (center) and angles (right) are shown for the single oligomer chain simulations (blue) and averaged over the 15 oligomers (red). S12

3.4 Radial distribution functions S13

S6 Radial Distribution Function (RDF) of (a,c) single oligomer chain and (b,d) averaged over 15 chains in (a,b) water:ethanol mixture and in (c,d) chloroform. The number of molecules is also shown as dashed lines. The colored shaded areas correspond to intervals between maximum and minimum values.S13

3.5 Contacts S14

S7 Absolute number of contacts for (a-d) single oligomer and (e-l) averaged over the 5 oligomers. The threshold of 4.5 Å was employed as the largest distance for establishing a contact. S14

S8 Schematic representation of spheres with 4.5 Å centered on different atoms of a representative PTQ(8bO2) configuration. S15

4 Dendrograms from hierarchical clustering **S16**

S9 Dendrogram from the clustering of PTQ(8bO2) in (a) aqueous mixture, (b) in chloroform, (c) PTQ10, (d) PTQ(C17) and (e) PTQ(8bO2) π-π stacking configurations. Each color corresponds to a different cluster and the dashed blue line is the RMSD threshold. S16

5 QM calculations **S17**

5.1 Benchmark of exchange-correlation functionals S17

S1 Excitation energy and oscillator strength of the first excited state (S_1) at different levels of theory for 1 up to 6 backbone units. Calculations were performed using the [1] 6-31G(d,p) and [2] def2-SVP atomic basis sets. OT-SRSH-PCM1 and OT-SRSH-PCM2 considered n-octanol ($\epsilon_1 = 9.86$) and 1-FluoroOctane ($\epsilon_2 = 3.89$), respectively. S17

5.2 Fragment-based analysis of excited states S18

S10 Fragment-based analysis of the medoid from cluster 1 of PTQ(8bO2) solvated in chloroform. (a) Excitation energies (black horizontal bars) and oscillator strengths (color plot) are shown in the top panel along with the excited state character of the first 25 singlet states in the bottom panel. (b) Representation of PTQ(8bO2) monomer divided into 3 fragments. (c) Quantum region comprising the 5 central monomers of the medoid from cluster 1. . . . . S18
S11 Fragment-based analysis of excited states of single monomer of PTQ(8bO2) at the (a) RI-SOS-PBE-QIDH/def2-SVP, (b) CAM-B3LYP/6-31G(d,p), (c) B3LYP/6-31G(d,p) and (d) M06/6-31G(d,p) levels of theory. S19
5.3 Atomic basis set dependency S20
S12 Absorption spectrum of a representative π-π stacking configuration computed with B3LYP (upper panel) and CAM-B3LYP (lower panel) using 6-31G(d,p), 6-311G(d,p), 6-31+G(d,p) and 6-311+G(d,p) atomic basis sets. The dimer spectrum is also compared with each isolated oligomer chain. . . S20
5.4 PCM absorption spectra S21
S13 Absorption spectra of the medoid from each cluster of PTQ(8bO2) stacking configurations. The spectrum computed for the interface (solid lines) is compared with the separated oligomers (dashed and dotted lines) solvated in the aqueous mixture (left panels) and in chloroform (right panels). . . . S21

6 Instruments and measurements **S22**

1 Oligomer structures and force field parameterization

Since the oligomers are formed by ten monomers, the number of atoms per polymer chain ranges from 702 for PTQ10 to 842 for PTQ(8bO2), making the geometry optimization unfeasible at the Density Functional Theory (DFT) level. Therefore, we optimized each polymer's monomer and trimer geometries and constructed the oligomer by bonding three trimers with a final monomer. The dihedral angles comprising the single bond between quinoxaline and thiophene units were adjusted to reproduce the values obtained for the trimer. All geometry optimizations were performed at the M06/6-311G(d,p) level of theory with the Polarizable Continuum Model (PCM) using n-Octanol as a solvent for PTQ(8bO2) and 1-FluoroOctane for PTQ10/PTQ(C17), to match the dielectric constant values measured for the materials, as proposed by Wang *et al.* [1].

For the force field parameterization, we computed partial charges at the same level of theory for the complete oligomer, only changing the solvent according to the simulation. In particular, the effective dielectric constant was determined with the Bruggeman model for simulations in the water and ethanol mixture. For the 15:85 v/v rate of water:ethanol, the effective dielectric constant is $\epsilon = 30.0805$, while the remaining solvent parameters were chosen as the ethanol values (see Gaussian16 manual).

Since single bonds can be flexible, we performed a rigid scan of the trimer's torsional angles between quinoxaline (Qx) and thiophene units. Side chains were removed during the scan and the constants that define the Ryckaert-Bellemans dihedral were fitted to reproduce the DFT energy profile. To avoid the multicollinearity problem due to the high correlation between dihedrals, Lasso regression with cross-validation was employed for the fitting, as implemented in the *scikit-learn* Python library [2]. The resulting classical energy barriers are compared to the quantum barriers in Fig. S1.

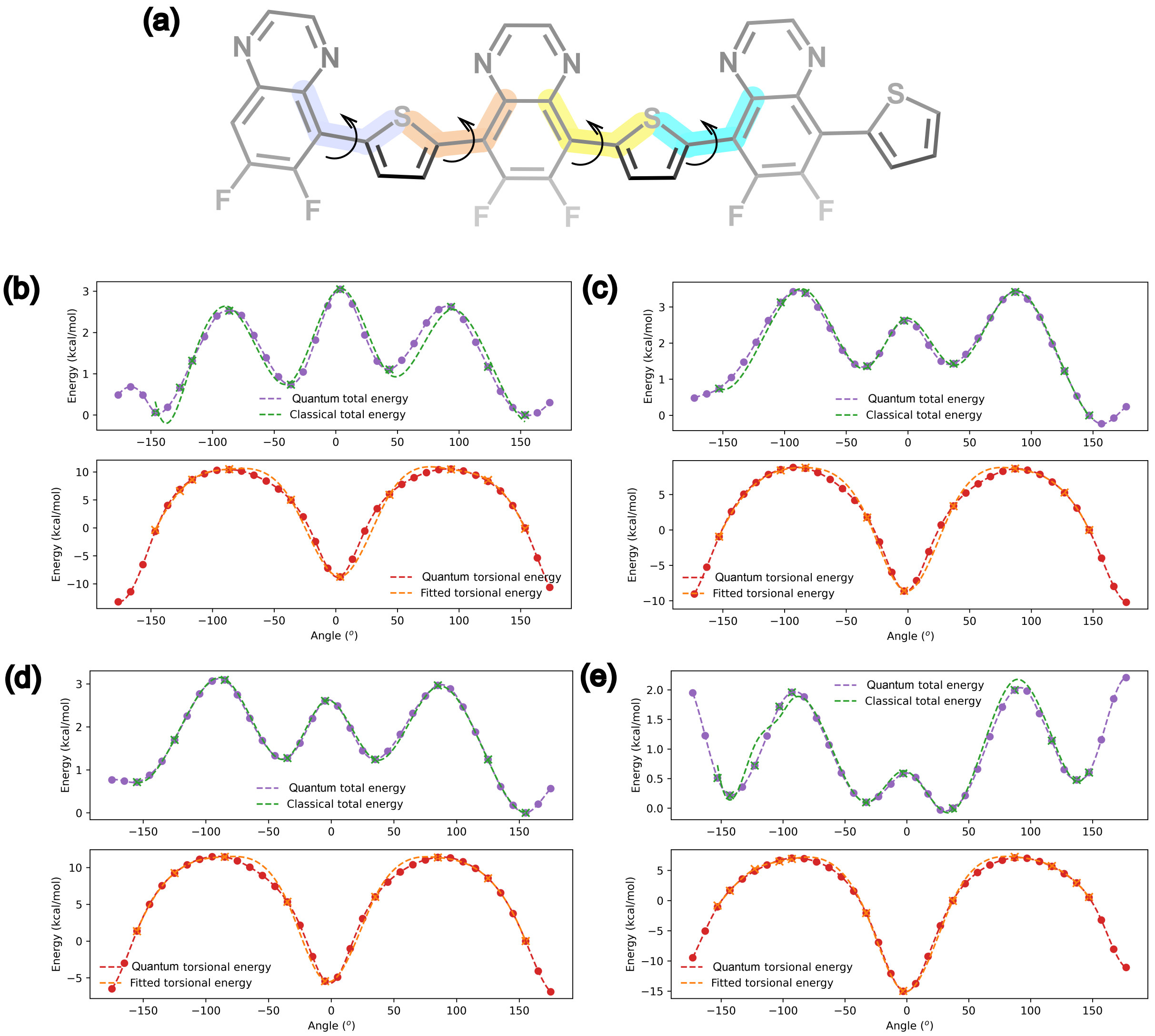

Figure S1: Energy profile of dihedrals fitted from rigid scan. (a) 2D representation of the trimer with fitted dihedrals highlighted. (b-e) Total (top panel) and torsional (lower panel) energies as a function of the dihedral angle. Each plot respectively corresponds to the energy profiles of the angles highlighted in (a), from left to right.

2 H link-atom method

Given the large number of atoms in each oligomer chain, we selected part of the molecule to be included in the quantum region and used the H link-atom method to saturate the bonds that cross the boundary between classical and quantum regions. As discussed throughout the Results section, we considered different quantum regions depending on the purpose and in all cases, we saturated C-C single bonds with hydrogen atoms. As recommended by Truhlar *et al.* [3], the hydrogens were added along the former bond at a distance corresponding to 0.71 times the original C-C distance.

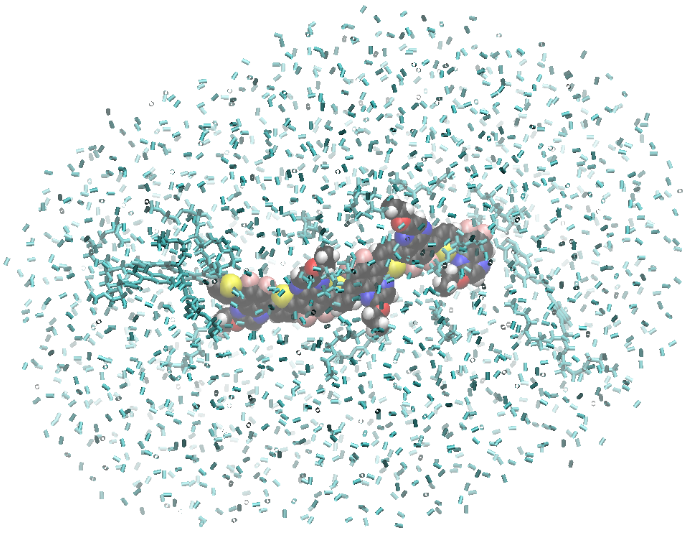

Figure S2: Representative configuration of a quantum region (vdW spheres) defined as 5 monomer backbones with all other atoms closer than 30 nm described as partial charges. This configuration was extracted from the simulation of a single oligomer chain of PTQ(8bO2) in chloroform.

For the calculations with point charges, we removed the point charge corresponding to the carbon bonded to the carbon in the quantum region. The net charge was then redistributed among the three closest neighbors. Then, the quantum region was centralized in the MD simulation box, and we considered all point charges up to 30 nm from the atoms, forming an electrostatic embedding shaped according to the geometry of the quantum system. Figure S2 illustrates the approach for a quantum region comprising five monomers without side chains.

Finally, the overall net charge was equally distributed over the 30% furthest atoms (typically more than 9000 atoms) to neutralize the system. This procedure was performed using the `prep_qmmm.py` tool that is available on GitHub.

3 MD simulations

3.1 Diffusion coefficients

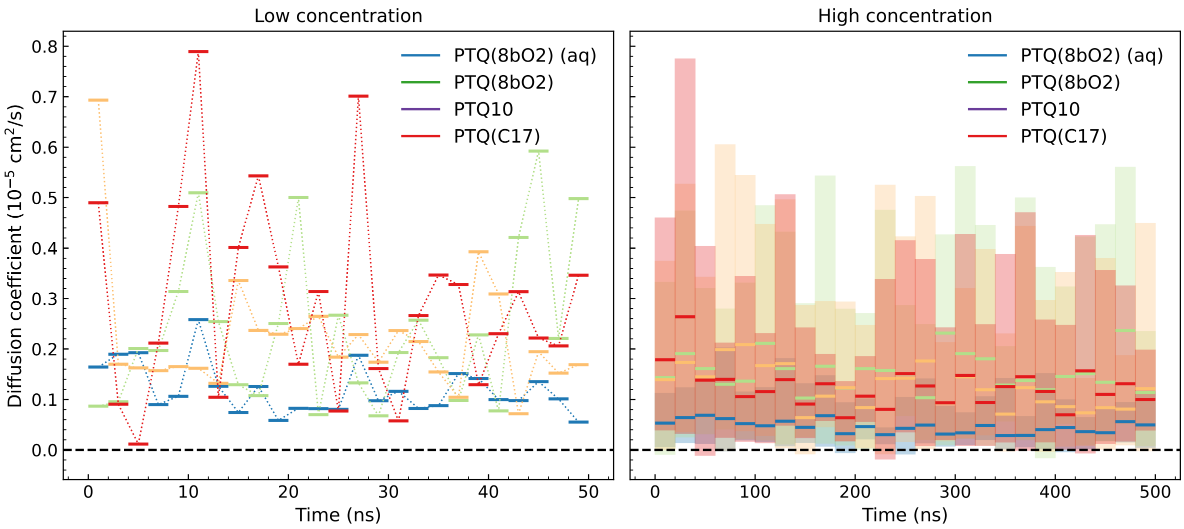

Figure S3: Time evolution of diffusion coefficients from single oligomer simulations (left) and averaged over the 15 oligomers from the 3 boxes with 5 oligomers each (right). Vertical bins in the right plot correspond to the variation between maximum and minimum values.

3.2 Distance matrix plot

The distance matrix plot is determined by computing the Euclidean distance between two sets of atoms and showing it using a color gradient. In our case, we selected a snapshot from one of the simulations of PTQ(8bO2) in chloroform at high concentration and considered all atoms belonging to the five oligomer chains. The snapshot is shown in Fig. S4(a), with each oligomer chain shown using the licorice representation and separated by color. Chloroform molecules are represented via black lines. Resonant structures are shown in Fig. S4(b) to improve the visualization of oligomer stacking.

Before computing the distances, we reordered the atom indexes so that all backbone atoms are together, followed by the atoms belonging to side chains. In Fig. S4(c), we present the distance plot with all 4210 atoms from all 5 oligomer chains, indicating the sets of backbone and side chains atoms inside the larger squares with the size of each oligomer. The main diagonal of larger squares corresponds to the distances between atoms belonging to the same oligomer chain, while the other squares cover different chains. Since two pairs of oligomer chains are at close distances, *i.e.*, brown is close to yellow, and orange is close to gray, we obtain four bright large squares out of the main diagonal. By zooming into the squares mapping oligomers 2 and 3, which, due to the symmetry, are either in the second column and third row or in the second row and third column, we have a better resolution of the atomic distances, as shown in Fig. S4(d). In this plot, the dashed black lines separate the stacking between the different monomers, allowing a better interpretation of the aggregation type.

A more straightforward interpretation is obtained by considering a threshold for typical π-π stacking distances. As shown in Fig. S4(e), light blue maps the atoms closer to 5 Å, while darker blue maps all other distances, which allows us to observe the stacking between specific monomers. Since the bright spots are deviating from the main diagonal, we can infer that the oligomers are not directly stacked in an H-type aggregation, and it suggests a mixed stacking more compatible with the J-type aggregation.

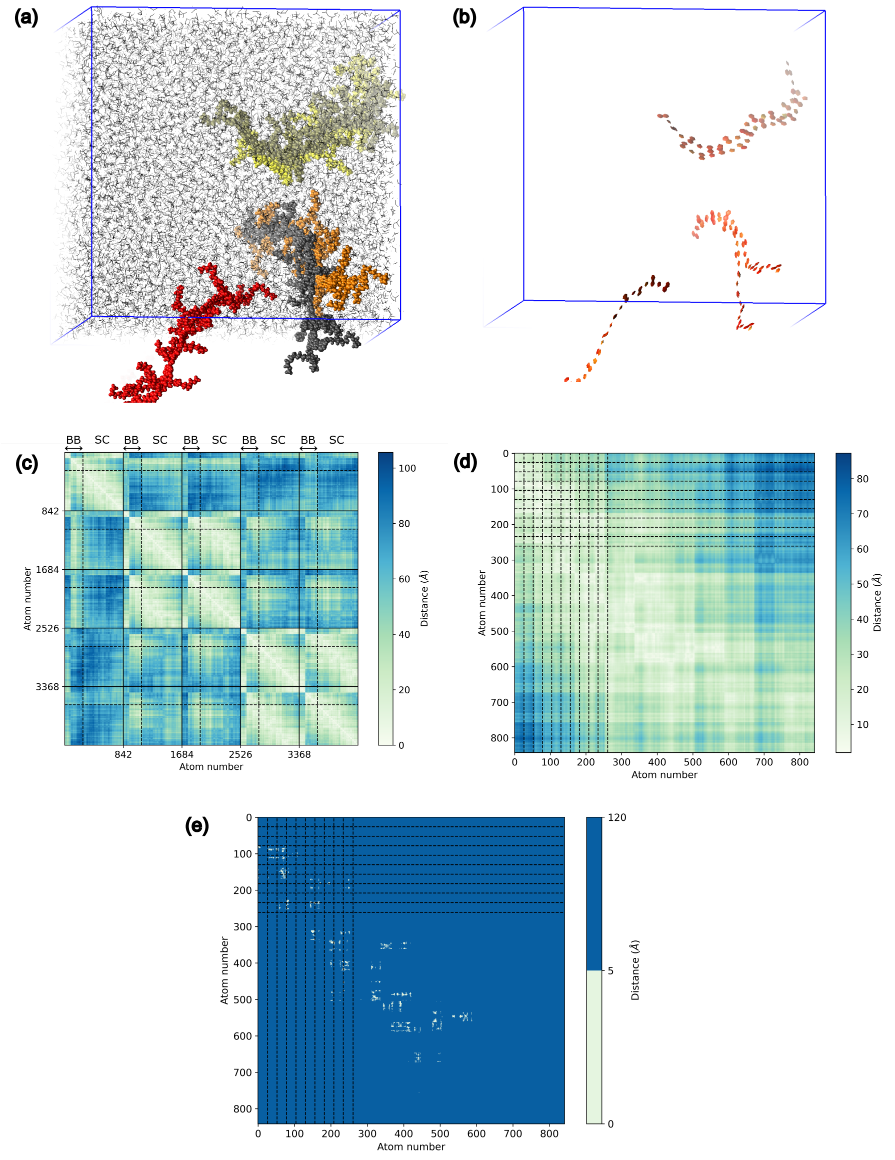

Figure S4: Aggregation of a representative snapshot from one of the simulations of 5 PTQ(8bO2) oligomer chains in chloroform. Snapshot of the (a) complete system and (b) only showing the resonant structures from backbones. Distance matrix plots (c) between all oligomer atoms and (d-e) between atoms from oligomers 2 and 3 that are aggregated.

3.3 Hydrogen bonds

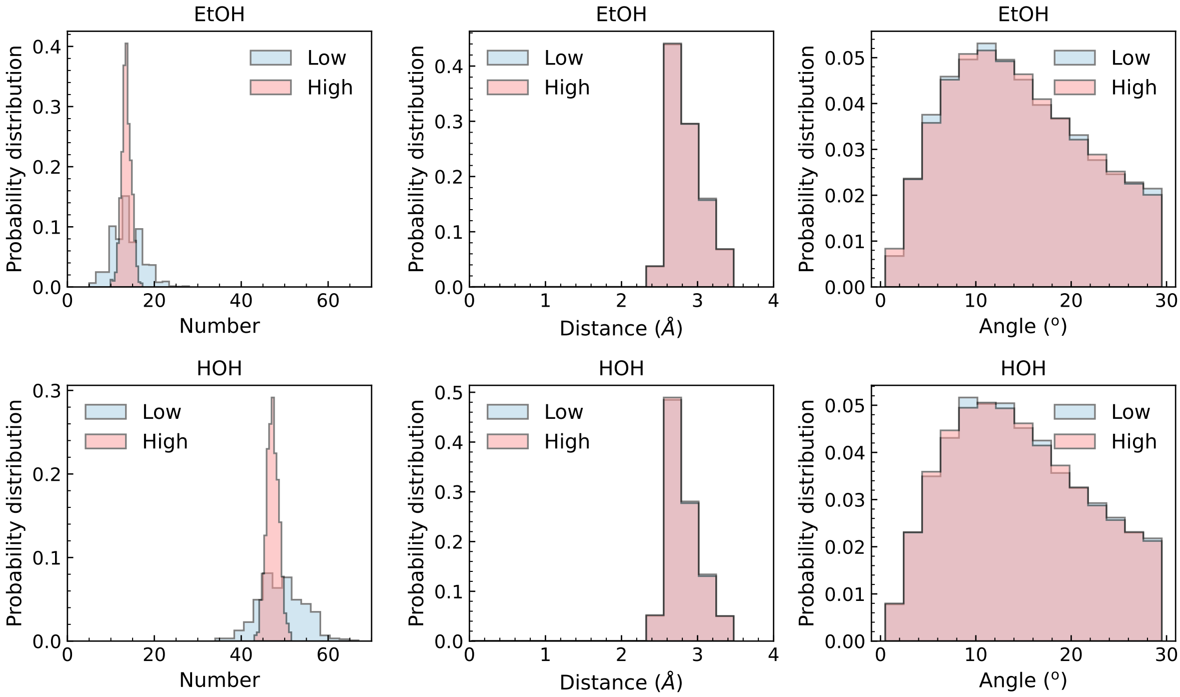

Figure S5: Hydrogen bonds of PTQ(8bO2) in water:ethanol mixture. Distribution of number of bonds (left), distances (center) and angles (right) are shown for the single oligomer chain simulations (blue) and averaged over the 15 oligomers (red).

3.4 Radial distribution functions

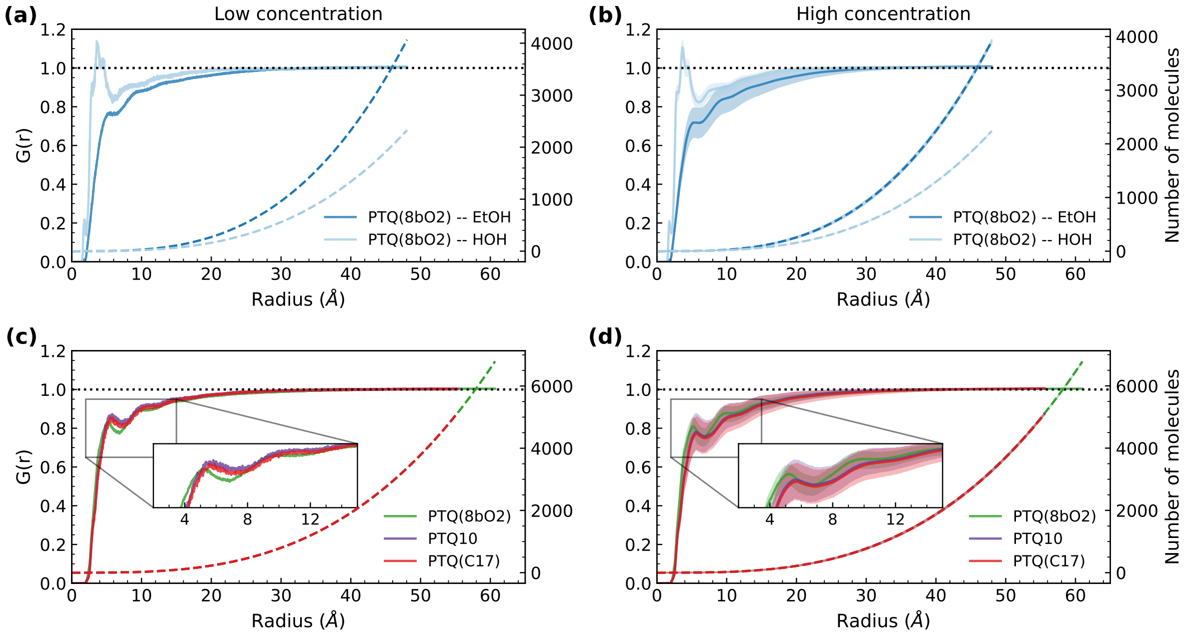

Figure S6: Radial Distribution Function (RDF) of (a,c) single oligomer chain and (b,d) averaged over 15 chains in (a,b) water:ethanol mixture and in (c,d) chloroform. The number of molecules is also shown as dashed lines. The colored shaded areas correspond to intervals between maximum and minimum values.

3.5 Contacts

In Fig. S7, the threshold of 4.5 Å was employed and the sum of intra and intermolecular contacts is presented as a function of time. Despite having the similar trend of Fig. 5, when considering the number of atoms in side chains and backbones, the differences between each curve are shifted, providing a more direct picture of the packing.

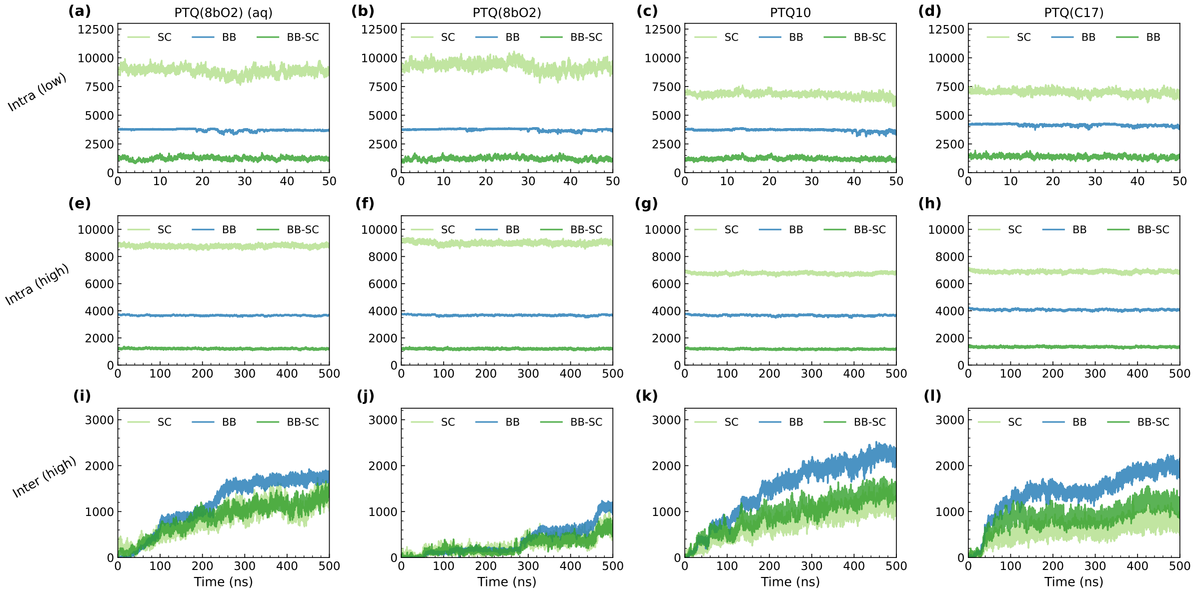

Figure S7: Absolute number of contacts for (a-d) single oligomer and (e-l) averaged over the 5 oligomers. The threshold of 4.5 Å was employed as the largest distance for establishing a contact.

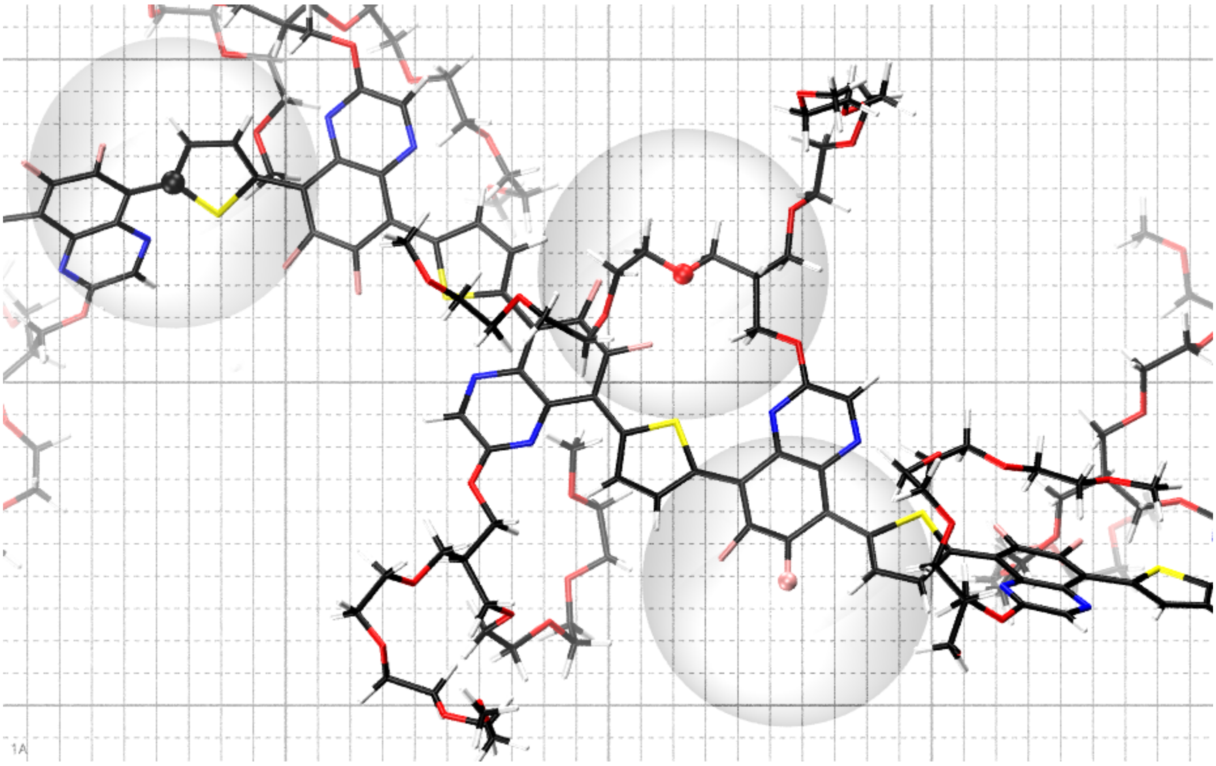

Figure S8: Schematic representation of spheres with 4.5 Å centered on different atoms of a representative PTQ(8bO2) configuration.

4 Dendrograms from hierarchical clustering

The dendrograms of single oligomer chains were generated from the hierarchical clustering scheme with average linkage and the RMSD threshold of 8.0 Å. For the clustering of π-π stacking configurations, the threshold was 5.0 Å.

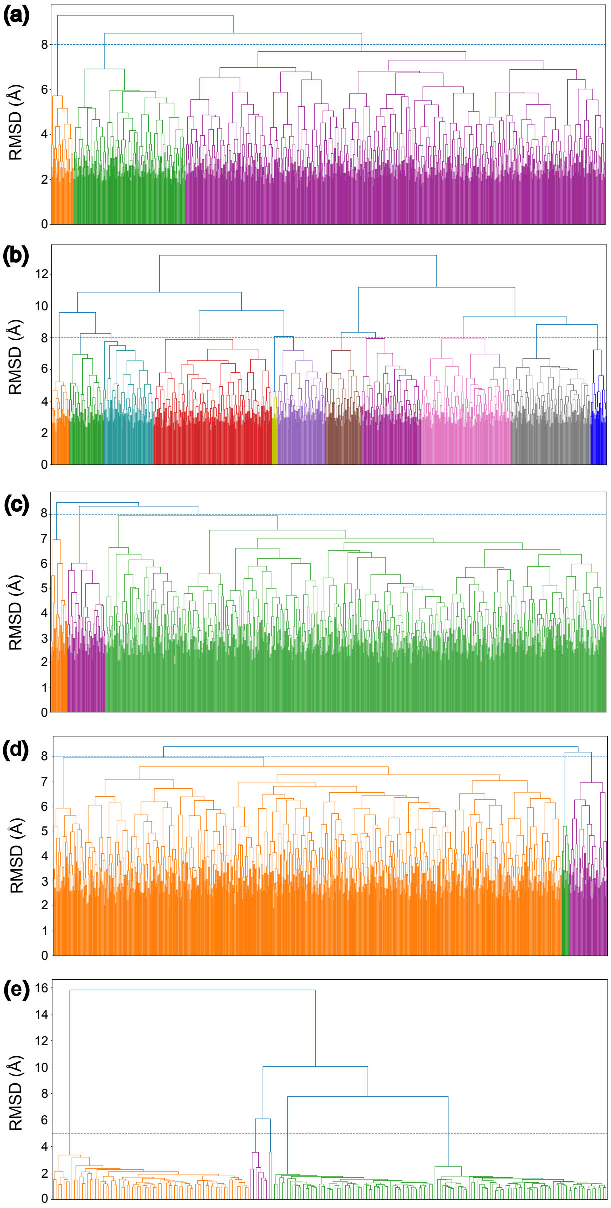

Figure S9: Dendrogram from the clustering of PTQ(8bO2) in (a) aqueous mixture, (b) in chloroform, (c) PTQ10, (d) PTQ(C17) and (e) PTQ(8bO2) π-π stacking configurations. Each color corresponds to a different cluster and the dashed blue line is the RMSD threshold.

5 QM calculations

5.1 Benchmark of exchange-correlation functionals

Number of monomers	Method	Energy (eV)	Wavelength (nm)	Oscillator strength
	M06[1]	3.42	362.91	0.13
	OT[1]	3.92	316.37	0.22
	OT-SRSH-PCM1[1]	3.88	319.71	0.31
1	OT-SRSH-PCM2[1]	3.88	319.72	0.30
	B3LYP[1]	3.33	372.60	0.11
	CAM-B3LYP[1]	3.91	316.72	0.23
	RI-SOS-PBE-QIDH[2]	4.13	300.50	0.37
	M06[1]	2.76	449.12	0.67
	OT[1]	3.08	402.90	0.81
	OT-SRSH-PCM1[1]	3.05	406.44	1.04
2	OT-SRSH-PCM2[1]	3.05	407.14	1.02
	B3LYP[1]	2.68	463.38	0.62
	CAM-B3LYP[1]	3.21	386.43	0.94
	RI-SOS-PBE-QIDH[2]	3.40	364.40	1.32
	M06[1]	2.55	486.34	1.19
	OT[1]	2.83	437.89	1.37
	OT-SRSH-PCM1[1]	2.81	440.66	1.64
3	OT-SRSH-PCM2[1]	2.81	441.67	1.61
	B3LYP[1]	2.45	507.00	1.10
	CAM-B3LYP[1]	3.01	412.41	1.55
	RI-SOS-PBE-QIDH[2]	3.17	390.90	1.97
	M06[1]	2.41	514.99	1.81
	OT[1]	2.67	465.06	2.05
	OT-SRSH-PCM1[1]	2.65	467.27	2.32
4	OT-SRSH-PCM2[1]	2.65	468.39	2.29
	B3LYP[1]	2.30	540.04	1.65
	CAM-B3LYP[1]	2.87	432.74	2.28
	RI-SOS-PBE-QIDH[2]	3.02	410.80	2.72
	M06[1]	2.32	534.49	2.15
	OT[1]	2.57	483.27	2.40
	OT-SRSH-PCM1[1]	2.56	484.38	2.64
5	OT-SRSH-PCM2[1]	2.55	485.63	2.61
	B3LYP[1]	2.20	562.75	1.97
	CAM-B3LYP[1]	2.78	445.93	2.65
	M06[1]	2.29	541.21	2.57
	OT[1]	2.54	487.72	2.90
	OT-SRSH-PCM1[1]	2.54	487.27	3.14
6	OT-SRSH-PCM2[1]	2.54	488.65	3.11
	B3LYP[1]	2.17	571.37	2.33
	CAM-B3LYP[1]	2.76	449.63	3.19

Table S1: Excitation energy and oscillator strength of the first excited state (S_1) at different levels of theory for 1 up to 6 backbone units. Calculations were performed using the [1] 6-31G(d,p) and [2] def2-SVP atomic basis sets. OT-SRSH-PCM1 and OT-SRSH-PCM2 considered n-octanol ($\epsilon_1 = 9.86$) and 1-FluoroOctane ($\epsilon_2 = 3.89$), respectively.

5.2 Fragment-based analysis of excited states

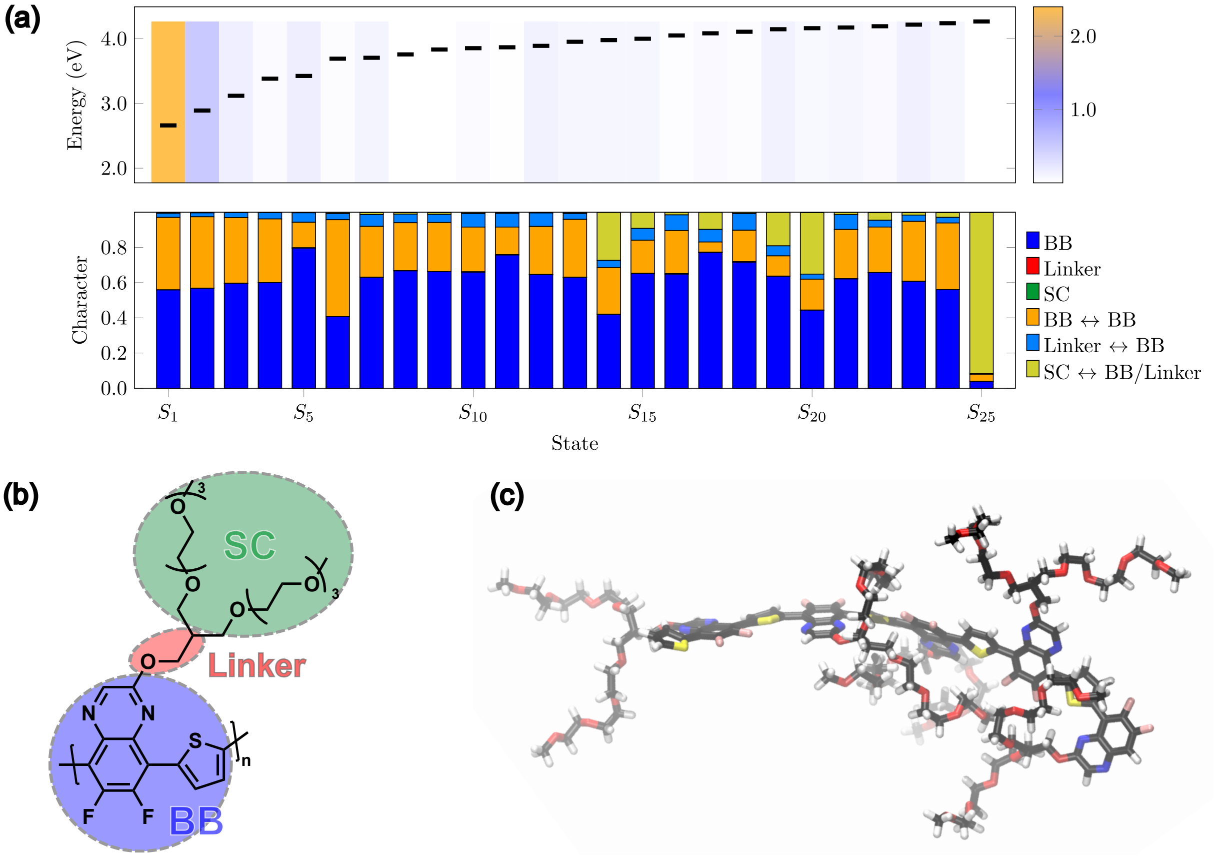

Figure S10: Fragment-based analysis of the medoid from cluster 1 of PTQ(8bO2) solvated in chloroform. (a) Excitation energies (black horizontal bars) and oscillator strengths (color plot) are shown in the top panel along with the excited state character of the first 25 singlet states in the bottom panel. (b) Representation of PTQ(8bO2) monomer divided into 3 fragments. (c) Quantum region comprising the 5 central monomers of the medoid from cluster 1.

To investigate the excitation character dependency over the XC functional, we computed the excited states of a single monomer with side chains explicitly included in the QM region. Instead of focusing on the linker group contribution, as done in Fig. S10, we divided the monomer into three regions comprising the atoms from difluoroquinoxaline (DFQ), thiophene (T) and side chain (SC) units. Here, the side chains also include the atoms from the linker group, shown in Fig. S10(b). The results are presented in Fig. S11.

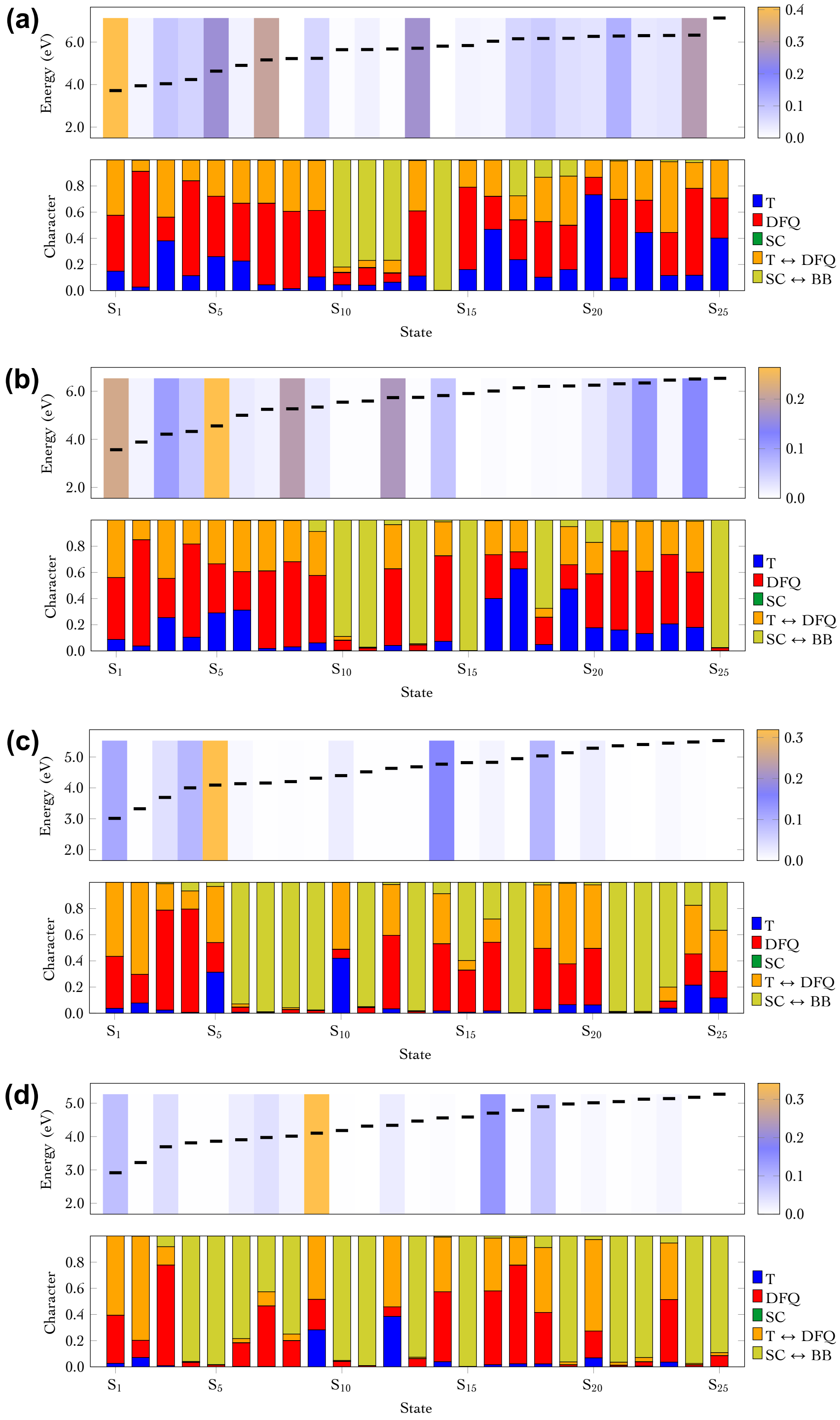

Figure S11: Fragment-based analysis of excited states of single monomer of PTQ(8bO2) at the (a) RI-SOS-PBE-QIDH/def2-SVP, (b) CAM-B3LYP/6-31G(d,p), (c) B3LYP/6-31G(d,p) and (d) M06/6-31G(d,p) levels of theory.

5.3 Atomic basis set dependency

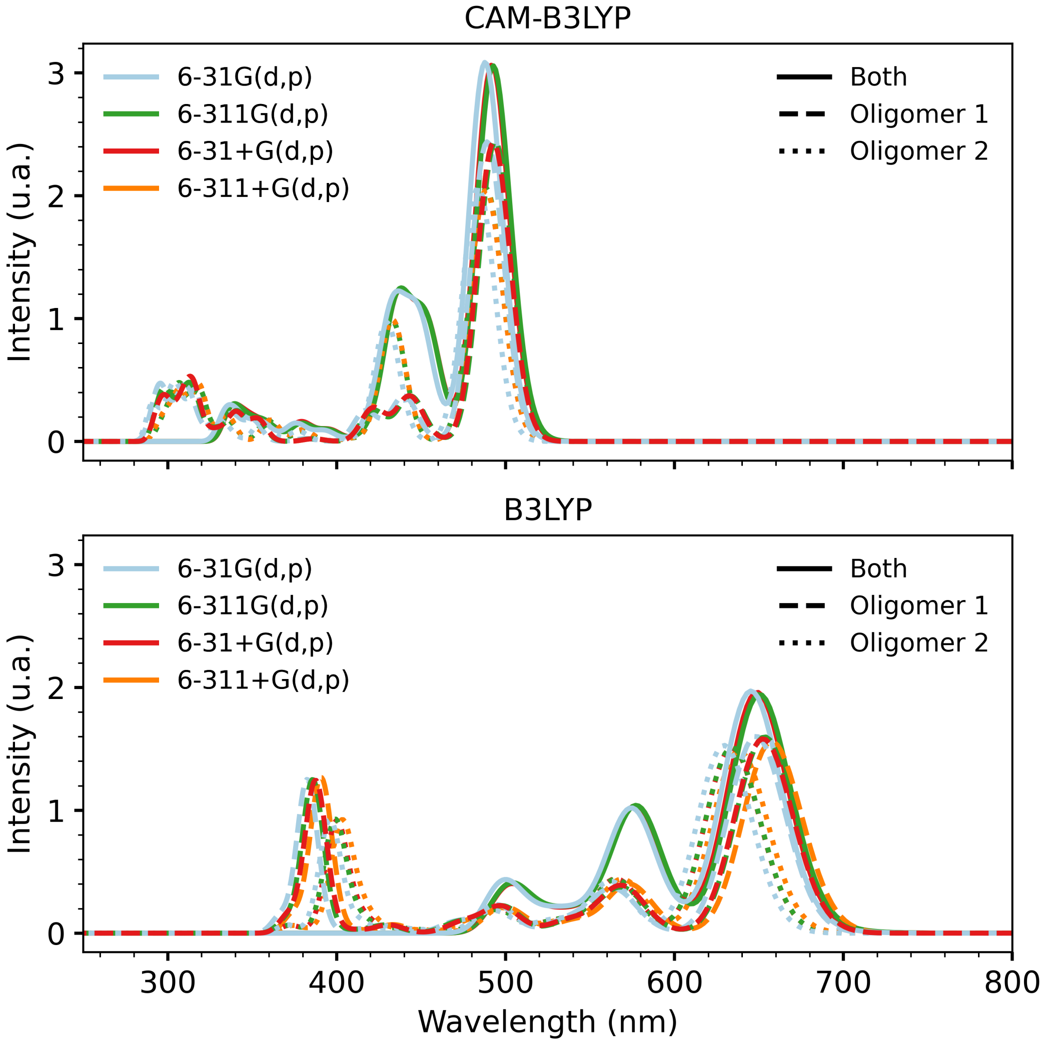

Figure S12: Absorption spectrum of a representative π-π stacking configuration computed with B3LYP (upper panel) and CAM-B3LYP (lower panel) using 6-31G(d,p), 6-311G(d,p), 6-31+G(d,p) and 6-311+G(d,p) atomic basis sets. The dimer spectrum is also compared with each isolated oligomer chain.

5.4 PCM absorption spectra

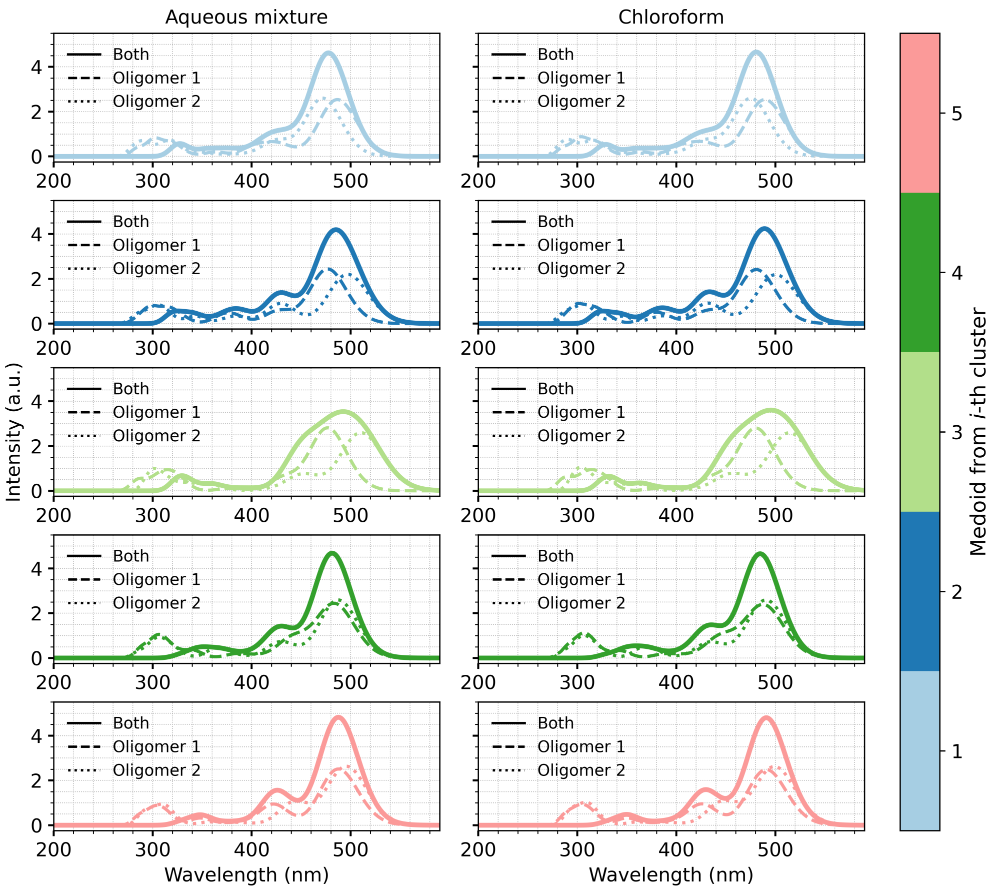

Figure S13: Absorption spectra of the medoid from each cluster of PTQ(8bO2) stacking configurations. The spectrum computed for the interface (solid lines) is compared with the separated oligomers (dashed and dotted lines) solvated in the aqueous mixture (left panels) and in chloroform (right panels).

6 Instruments and measurements

PTQ10 and PTQ(8bO2) were synthesized accordingly to reported procedures in references [1] and [4]. PTQ10 and PTQ(8bO2) were achieved with $M_n = 47$ kg mol^{-1} (Đ = 2.1) and $M_n = 16$ kg mol^{-1} (Đ = 2.2), as previously reported in reference [1]. The number-average molecular weight (M_n) and dispersity (Đ) of the polymers were determined by size-exclusion chromatography (SEC) analyses using an Agilent GPC 1200 instrument equipped with a refractive index detector. The system was calibrated with polystyrene standard, and ortho-dichlorobenzene (80 °C) was used as eluent.

Absorption spectra were recorded on a PerkinElmer lambda 1050 UV/vis/NIR spectrometer, using a fixed concentration in polymer of 0.03 mg mL^{-1}. For the preparation of the solutions, 1 mg of polymer was solubilized in 1 mL to constitute a stock solution of 1 mg mL^{-1}. The solution was heated up to 50 °C for 2 hours to ensure complete solubility of the materials. Once the solution cooled down to room temperature, 90 μL of the stock solution were diluted in 2.91 mL of solvent (*i.e.* $CHCl_3$ or water:ethanol mixture) to achieve a final concentration of 0.03 mg mL^{-1}. Considering that all materials are readily soluble in chloroform, the comparison of solubility properties remained linked to intrinsic properties rather than solubility issues, especially considering the low concentrations used for preparation and characterization. In the case of PTQ(8bO2), it remains highly soluble higher concentrations in water:ethanol ($>$ 10 mg mL^{-1}, as reported in reference [1]), therefore, the low concentration used for comparison was not at the limit of solubility and is more related to the polymer's property.

References

(1) T. T. Filate, S. Lee, L. R. Franco, Q. Chen, Z. Genene, C. F. N. Marchiori, Y. Lee, M. Araujo, W. Mammo, H. Y. Woo, B. J. Kim and E. Wang, *ACS Applied Materials & Interfaces*, 2024, **16**, 12886–12896.

(2) F. Pedregosa, G. Varoquaux, A. Gramfort, V. Michel, B. Thirion, O. Grisel, M. Blondel, P. Prettenhofer, R. Weiss, V. Dubourg et al., *Journal of Machine Learning Research*, 2011, **12**, 2825–2830.

(3) H. Lin and D. G. Truhlar, *The Journal of Physical Chemistry A*, 2005, **109**, 3767–4016.

(4) J. J. Rech, J. Neu, Y. Qin, S. Samson, J. Shanahan, R. F. J. III, H. Ade and W. You, *ChemSusChem*, 2021, **14**, 3561–3568.
